# External auditory exostoses among western Eurasian late Middle and Late Pleistocene humans

**DOI:** 10.1371/journal.pone.0220464

**Published:** 2019-08-14

**Authors:** Erik Trinkaus, Mathilde Samsel, Sébastien Villotte

**Affiliations:** 1 Department of Anthropology, Washington University, Saint Louis, MO, United States of America; 2 UMR5199 PACEA, Université de Bordeaux, Bâtiment B8, Allée Geoffroy Saint Hilaire, Pessac, France; 3 CNRS, UMR5199 PACEA, Bâtiment B8, Allée Geoffroy Saint Hilaire, Pessac, France; Max Planck Institute for the Science of Human History, GERMANY

## Abstract

External auditory exostoses (EAE) have been noted among the Neandertals and a few other Pleistocene humans, but until recently they have been discussed primary as minor pathological lesions with possible auditory consequences. An assessment of available western Eurasian late Middle and Late Pleistocene human temporal bones with sufficiently preserved auditory canals (n = 77) provides modest levels of EAE among late Middle Pleistocene archaic humans (≈20%) and early modern humans (Middle Paleolithic: ≈25%; Early/Mid Upper Paleolithic: 20.8%; Late Upper Paleolithic: 9.5%). The Neandertals, however, exhibit an exceptionally high level of EAE (56.5%; 47.8% if two anomalous cases are considered normal). The levels of EAE for the early modern humans are well within recent human ranges of variation, frequencies which are low for equatorial inland and high latitude samples but occasionally higher elsewhere. The Early/Mid Upper Paleolithic frequency is nonetheless high for a high latitude sample under interpleniglacial conditions. Given the strong etiological and environmental associations of EAE development with exposure to cold water and/or damp wind chill, the high frequency of EAE among the Neandertals implies frequent aquatic resource exploitation, more frequent than the archeological and stable isotopic evidence for Middle Paleolithic/Neandertal littoral and freshwater resource foraging implies. As such, the Neandertal data parallel a similar pattern evident in eastern Eurasian archaic humans. Yet, factors in addition to cold water/wind exposure may well have contributed to their high EAE frequencies.

## Introduction

In his classic monograph on the Neandertal skeleton from the Bouffia Bonneval in La Chapelle-aux-Saints, Corrèze, France, Marcellin Boule [1] noted the presence of bony growths (exostoses) in the auditory canals (“Ces orifices présentent quelques exostoses. L’orifice gauche est rétréci vers son milieu par des productions osseuses qui lui donnent une forme en sablier” ([1] p. 152). He did not comment further on them, but since that time the presence of external auditory exostoses has been noted in ten Middle Pleistocene humans [2–5], three additional Neandertals [6,7], three eastern Eurasian later archaic human [5], a few eastern early modern humans [5,8], and in one western Upper Paleolithic modern human [9]. Given that the frequencies of these bony growths across recent human populations appear to reflect primarily patterns of environmental exposure (plus inherited predispositions to form them) [10], an assessment of them among western Eurasian Late Pleistocene humans may provide insights into the morbidities and (by extension) behaviors of late archaic to early modern humans in the region.

External auditory exostoses (EAE; torus acusticus) are dense bony growths protruding into the external auditory canal. They are frequently observed clinically in modern humans, often in the context of aquatic sports (hence references to their presence as “swimmer’s ear” or “surfer’s ear”). First documented more than a century ago clinically [11,12], their distributions across human populations have been considered anthropologically since the 1930s [13–17], being employed as one of suite of discrete cranial traits for population affinity studies. However, their distribution among later Holocene humans has been shown to vary latitudinally and to correlate with habitual exposure to cold water [5,18–21]. In particular, clinical and bioarcheological observations indicate that they are principally due to an environmental irritation of the mucoperiosteum of the external auditory canal (see [10,18,22]). As first noted by Van Gilse [23] and Harrison [24], the most frequently observed irritant is cold water, in the context of cold water sports [25–31] or foraging [19,21,32–34]. Clinical reports also show that ears exposed to a combination of water and cold air, as represented by wind chill, are more likely to develop EAE and at a faster rate [25,28,35].

However, EAE may occur in a variety of contexts, given sufficient inflammation of the soft tissue lining of the auditory canal. They are often benign but can lead to cerumen impaction and conductive hearing loss [24,26,36–39] (see [7]). They are also frequently more common in males [14,18,21,40–44] (but see [32]), probably relating more to sex differences in activities than differential susceptibility [18,45].

Given this framework, observations on EAE among the available western Eurasian Middle and Upper Paleolithic humans, including both presence/absence and degree of formation, are provided. The primary samples of concern are Middle Paleolithic Neandertals and Upper Paleolithic modern humans, but observations are also included for the few sufficiently intact late Middle Pleistocene archaic humans and early Late Pleistocene (Middle Paleolithic) modern humans.

## Materials and methods

External auditory exostoses are identified as growths into the auditory canal from its squamous and/or tympanic portions [16,22,46]. They are usually rounded protrusions and may occur as single or multiple growths. They normally do not involve or development from the tympanosquamous or tympanomastoid sutures; such sutural protrusions are osteomata, or benign neoplasms, which normally occur laterally within the meatus, are less frequent and are often solitary [22,46].

The EAE were scored using the four part ordinal scale of Cooper et al. [26], Crowe et al. [41] and Villotte et al. [32] (see also [16]). Grade 0 indicates the absence of EAE, Grade 1 indicates small mildly protruding single or multiple exostoses (<1/3 of the canal), and Grade 2 reflects one or more large EAE projecting well into the auditory canal (1/3–2/3 of the canal). Grade 3 indicates a meatus which is largely or completely blocked by exostosis growth (>2/3 of the canal). Given the irregularities of EAE growths and their variable positions with the canal versus at the lateral margin, these grade distinctions are inevitably subjective but nonetheless serve to categorize EAE severity. For crania that provide bilateral observations of EAE, the EAE is scored as “present” if an exostosis is evident on at least one side. In the comparisons by grade, each side is scored as 0.5 in the sample summaries by grade if the two meatus have different grades of EAE formation.

A number of the Pleistocene tympanic bones are thickened at their lateral margins, especially where the inferior tympanic crest meets the porus, and some of them exhibit small nubbins of bone along the lateral tympanic margin. These changes are not considered to be part of the EAE, which consist of bony growths into the meatus.

The paleontological observations (S1 Table) have been made whenever possible visually on twenty-nine original Pleistocene specimens, by us (n = 24) and/or the paleontologists who have commented on presence/absence of EAE in individual specimens (n = 5). When high quality (resin) casts are available, they have been employed. Otherwise, the additional observations are based on high resolution digital scans of the temporal bones or detailed photographs that provide a clear view directly into the auditory meatus. For the lost Předmostí crania, observations are based on the sufficiently clear photographs in Velemínská and Brůžek [47]. The photographs and scans employed are available either as referenced in S1 Table or through the authors. The resin casts indicated are available in the Departments of Anthropology of either Washington University in Saint Louis or the University of Iowa, Iowa City. The observations of EAE presence/absence and degree of development, made on original fossil specimens by other scholars, are presented and referenced in S1 Table. The 24 original fossil specimens, on which we scored EAE and which are listed in S1 Table, are curated in the institutions provided in Table 1. Except for the Cro-Magnon and Pataud remains in the Musée de l’Homme (Colhelper request 46669), permissions were obtained through correspondence with the person(s) responsible for the fossil remains (Table 1).

**Table 1:** Locations of original fossil human specimens observed by us for the scoring of external auditory exostoses.

*Specimen*	*Curating Institution*	*Location*	*Permission*
Shanidar 1, 5	Iraq Museum	Baghdad, Iraq	Isa Salman
Bausu da Ture 2	Musée d’Archéologie Nationale	Saint-Germain-en-Laye, France	C. Schwab
Cro-Magnon 1, 2	Musée de l’Homme	Paris, France	D. Grimaud-Hervé, V. Laborde
Dolní Věstonice 13 to 16	Archeologický ústav AV CR	Dolní Vĕstonice, Czech Republic	J. Svoboda, S. Sázelová
Muierii 2	Institutul de Speologie "Emil Racoviţă"	Bucharest, Romania	S. Constantin
Oase 2	Institutul de Speologie "Emil Racoviţă"	Bucharest, Romania	S. Constantin
Pataud 1	Musée de l’Homme	Paris, France	D. Grimaud-Hervé, V. Laborde
Pavlov 1	Archeologický ústav AV CR	Dolní Vĕstonice, Czech Republic	J. Svoboda, S. Sázelová
Sunghir 1	Laboratory of Anthropological Reconstruction, Russian Academy of Sciences	Moscow, Russia	T.S. Balueva
Bichon 1	Laténium: Parc et Musée d'Archéologie de Neuchâtel	Hauterive, Switzerland	M.-A. Kayser, D. Ramseyer, F.-X. Chauvières
Chancelade 1	Musée d’Art et d’Archéologie du Périgord	Périgueux, France	V. Merlin-Anglade
Iboussières A	Musée d'Archéologie Tricastine, Service Régional d’Archéologie Rhône-Alpes	Saint-Paul-Trois-Châteaux, France	M. Lert
Lafaye 1	Musée d’Histoire Naturelle Victor Brun	Montauban, France	A. Berteret
Laugerie Basse 4	Musée d’Archéologie Nationale	Saint-Germain-en-Laye, France	C. Schwab
La Peyrat 5	Musée Labenche	Brive-la-Gaillarde, France	L. Michelin
Rochereil 1	Institut de Paléontologie Humaine	Paris, France	H. de Lumley
St. Germain-la-Rivière 4	Musée National de Préhistoire	Les Eyzies, France	J.-J. Cleyet-Merle
San Teodoro 1, 2	Museo Geologico Gemmellaro	Palermo, Italy	C. Di Patti

The principal methodological limit of the study is the difficulty in scoring early EAE (see [30,32]), and the relative subjectivity in the ordinal scoring an irregular continuous trait (i.e. the relative size of an exostosis into the auditory canal), especially considering the diversity of the material used (original fossils, casts, scans, photographs). In order to circumvent this issue, the grade scores allotted in the study were checked by all authors and discussed before reaching a final agreement. Moreover, if the EAE appears intermediate between two grades, it was counted conservatively as the lower grade. Thus, even if our study cannot be considered as fully reproducible by other researchers, we consider that this would produce only minor differences (i.e. differences between two adjacent grades for few individuals). For example, the differences between the Neandertal sample and the more recent ones are evident for each grade of EAE (see below), making it unlikely that small changes in some individual scores would alter the clear trend illustrated here.

The primary samples include Neandertals and earlier and later Upper Paleolithic modern humans (S1 Table). The Neandertal sample (n = 23) derives from Europe and southwest Asia, from early Marine Isotope Stage (MIS) 5 to the middle of MIS 3. The Upper Paleolithic modern humans are separated into an MIS 3 Early/Mid Upper Paleolithic (E/MUP) sample (83.3% MUP) (n = 24) and an MIS 2 Late Upper Paleolithic one (n = 21); the former sample is entirely European, but the latter one includes one southwest Asian specimen (Ohalo 2). Additional data are included for small samples of late Middle Pleistocene (MIS 6) archaic humans (n = 5) and an early Late Pleistocene (MIS 5) Middle Paleolithic modern humans (MPMH; n = 4).

The three primary samples are all male biased (for the sexable specimens) (Table 2), although they are not different from each other in sex proportions (χ^2^ p = 0.976). They are also biased towards young adults plus older adolescents versus older adults (Table 2), as are Late Pleistocene mortality profiles in general [48]. However, the three samples are not significantly different from each other if the adolescents and young adults are pooled (χ^2^ p = 0.941). Therefore, although EAE, as a degenerative process, generally increases in frequency and/or severity with age and/or exposure [30,34,43,49–51], the age distributions should not affect the comparisons across these Late Pleistocene samples.

**Table 2:** Sex and age distributions for the late Middle and late Pleistocene samples.

	Late Middle Pleistocene	Neandertal	Mid. Paleol. Mod. Humans	Early/Mid Upper Paleol.	Late Upper Paleolithic
Sex^1^					
Male	0	8	1	12	12
Female	0	4	1	7	7
Indeterminate	5	11	2	5	2
Age					
Adol./YA^2^	1	14	4	15	11
Old Adult	0	7	0	6	6
Adult	4	2	0	3	4

^1^ The male and female counts include both pelvically and craniofacially sexed remains.

^2^ Given that the adolescent remains are all late adolescent and routinely considered as adult, and that the included cranial/temporal remains from the large Krapina sample cannot be aged to adolescent versus young adult, the adolescent and young adult (YA) samples are pooled in the counts.

To provide a recent human reference framework, presence/absence data are presented for 120 recent human samples (S2 Table). As originally proposed by Kennedy [18], they are separated by latitude into those <30°, those between 30° and 45°, and those >45° (north and south). In addition, within each latitudinal range and given the predilection for EAE to form when the auditory canal is exposed to cold water, they are separated into “wet” versus “dry” samples (S2 Table); the former samples are coastal/riverine/lacustrine ones with known or likely exposure to water, and the latter are inland ones with little aquatic exposure. It is fully recognized that these “wet” and “dry” categories are approximate, given the archeological contexts of many of the samples. However, separating the samples along these lines provides additional detail for the recent human framework, given the differences in EAE frequencies evident between adjacent coastal and inland samples [19,33,34]; minor resorting of the samples into the “wet” versus “dry” categories is unlikely to alter substantially the distributions of presence/absence of EAE. Moreover, they are provided as a framework for evaluating the later Pleistocene sample frequencies and not as a reassessment of recent human EAE epidemiology. Only samples with n > 30 are included, and pooled national, large island and immigrant samples are excluded. Given the absence of male versus female and age data for many of the samples, sexes are pooled where frequencies are provided separately by sex and all are considered as “adult” unless otherwise indicated. There are 30 low-latitude, 15 mid-latitude and 16 high-latitude samples assigned to the ‘wet’ group, and 22 low-latitude, 19 mid-latitude and 18 high-latitude samples assigned to the ‘dry’ group (S2 Table).

In addition, to provide a framework for EAE severity distributions, the fossil scores are compared to the available grade data for a smaller number (14) of recent human samples [19,21,32,42,51,52], also divided into “wet” (n = 10) and “dry” (n = 4) categories with the frequencies pooled within each category. The resultant frequencies are grade 0: 98.2%, grade 1: 1.8%, grade 2: 0.0% and grade 3: 0.0% for the pooled “dry” sample; they are grade 0: 76.6%, grade 1: 16.6%, grade 2: 4.5% and grade 3: 2.4% for the pooled “wet” sample (S3 Table).

In the statistical comparisons, exact Chi-square and binomial p-values were computed using StatXact 4.0.1 [53], and Wilcoxon / Mann-Whitney p-values were determined using NCSS 11.0.19 [54] for the latitudinal distributions of the samples. Binomial 95% confidence intervals for the fossil sample EAE percentages derive from the online calculator of Soper [55].

## Results

### Presence/Absence of external auditory exostoses

**Fig 1:**
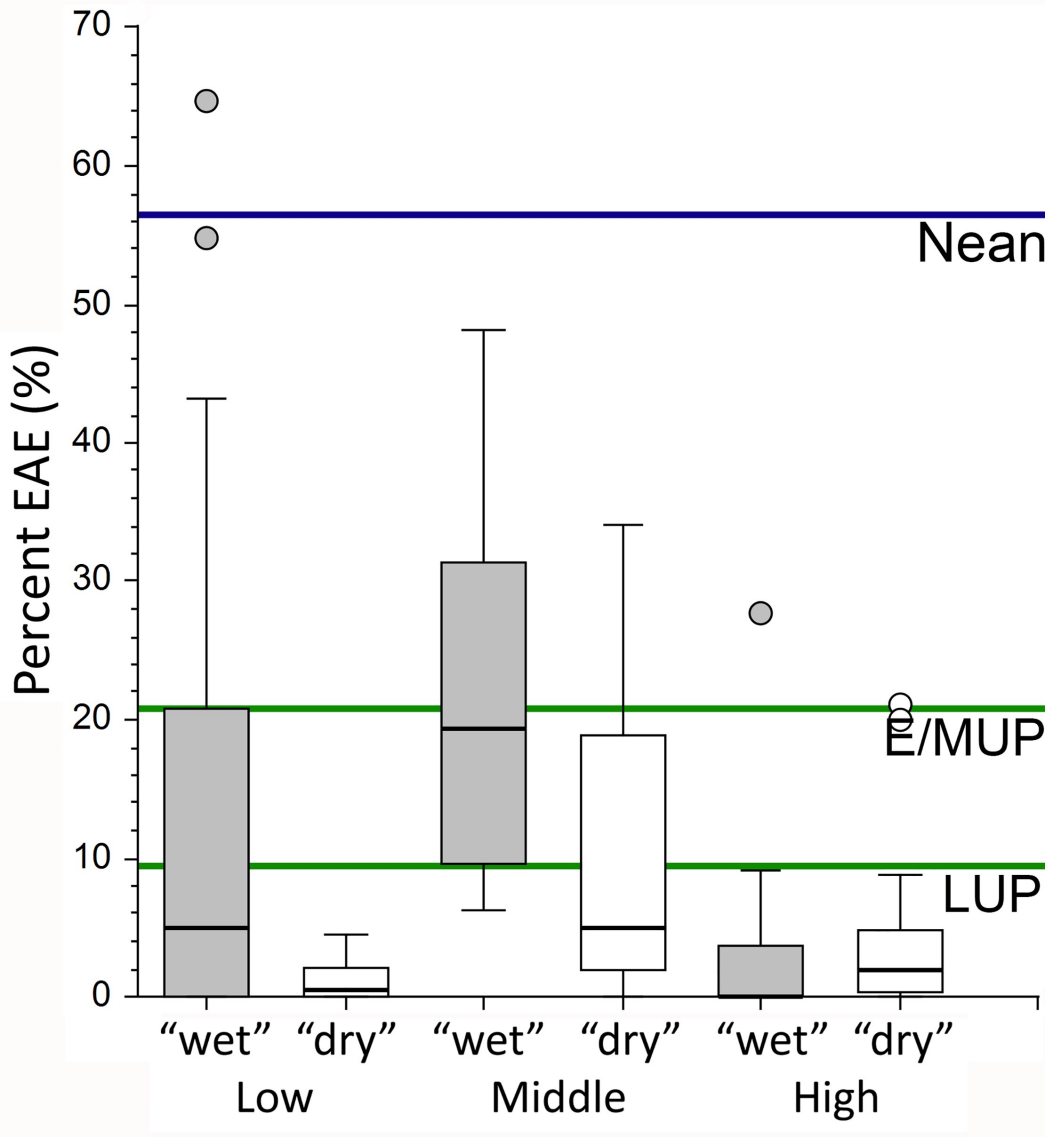
Percent presence of external auditory exostoses (EAE) in samples of Neandertals, Early/Mid Upper Paleolithic modern humans (E/MUP), Late Upper Paleolithic modern humans (LUP), and latitudinal samples of recent humans. The recent humans are divided into low (<30°), middle (35°– 40°) and high (>40°) latitude samples, and each one is then separated into coastal/riverine/lacustrine (“wet”) and inland (“dry”) samples; see S2 Table for justification and individual sample percentages. The 95% CIs for are 34.5%– 76.8% for the Neandertals, 4.8%– 48.5% for the E/MUP sample, and 1.2%– 30.4% for the LUP sample.

All of the later Pleistocene western Eurasian samples provide at least one individual with clearly developed external auditory exostoses. In the two smaller samples, the late Middle Pleistocene archaic human and the Middle Paleolithic modern human ones, one individual in each sample provides a modest (grade 1) EAE, providing sample frequencies of 20% and 25% (despite large 95% confidence intervals (CIs) of 5.1%– 71.6% for the late Middle Pleistocene sample and 6.3%– 80.6% for the Middle Paleolithic modern human one). If the values have any meaning given the large CIs, they suggest frequencies among the higher of the recent human middle latitude and low latitude “wet” samples (Fig 1).

The larger Upper Paleolithic samples provide frequencies with smaller 95% CIs, ones of 20.8% (CI: 4.8%– 48.5%) for the E/MUP sample and 9.5% (CI: 1.2%– 30.4%) for the LUP sample (Fig 1). Their 95% CIs span two-thirds of the recent human samples, but the mean values (especially the higher E/MUP one) are above most of the high latitude percentages and above all of the low latitude “dry” ones; yet they are well within the other recent human ranges of frequencies. The E/MUP frequency is nonetheless at or above the interquartile ranges of all of the recent human distributions except for the middle latitude “wet” sample.

In contrast, the Neandertal sample, with a mean frequency of 56.5% (CI: 34.5%– 76.8%) is well above all except two low latitude “wet” samples, one each from the Canary Islands and the southern Brazilian coast (S2 Table). The next closest are a middle latitude “wet” sample from eastern Turkey and low latitude “wet” one from the southern Brazilian coast [31,51,56]. Even its lower 95% CI limit is above the frequencies of all except six recent human samples (four low latitude “wet” ones and two middle latitude “wet” ones (S2 Table)), or 5.0% of the 120 recent human samples. The Neandertal frequency is significantly above the LUP one and its lower CI limit only overlaps the upper CI limit of the E/MUP sample modestly.

### Distributions of external auditory exostosis severity

**Fig 2:**
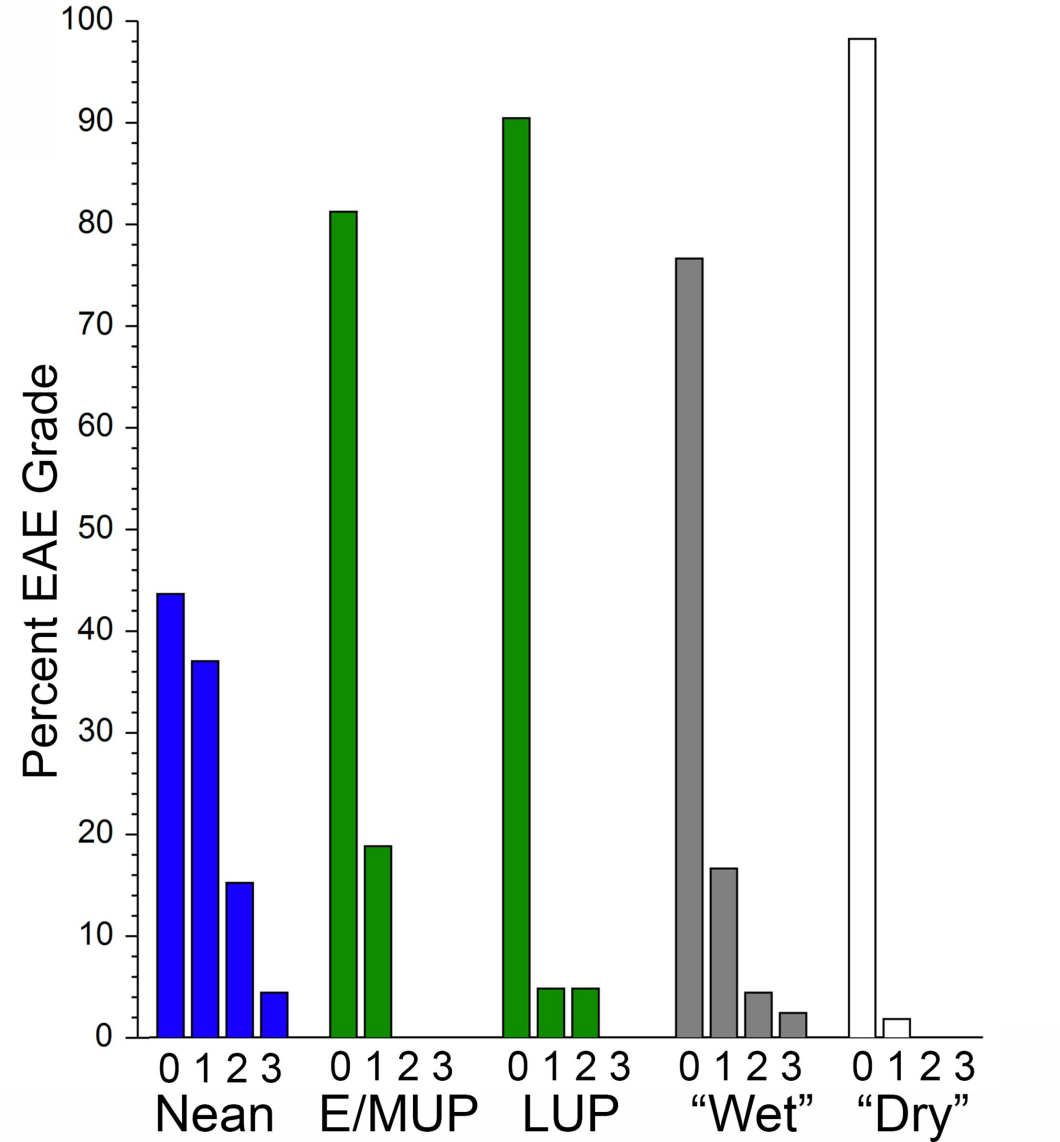
Distributions of external auditory exostosis (EAE) grades for samples of Neandertals, Early/Mid Upper Paleolithic modern humans (E/MUP), Late Upper Paleolithic modern humans (LUP), and coastal/riverine/lacustrine (“wet”) versus inland (“dry”) samples of recent humans. The paleontological data are in S1 Table, and the recent human data are in S3 Table.

**Fig 3:**
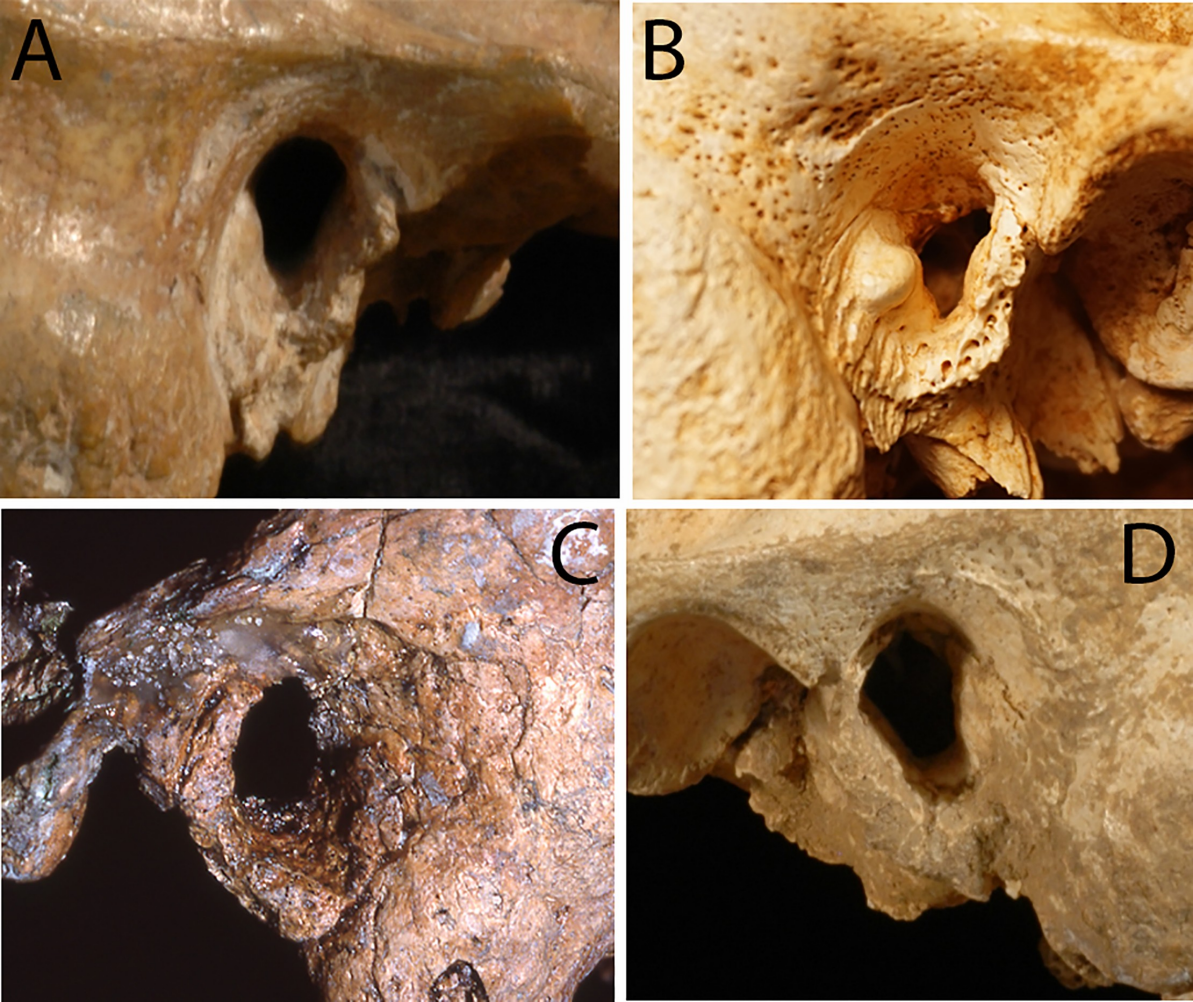
Examples of external auditory exostoses (EAE) among early modern human specimens. A: E/MUP Cioclovina 1 right (grade 1); B: LUP Iboussières A right (grade 2); C: MPMH Skhul 6 left (grade 1); D: E/MUP Oase 2 left (grade 1). Not to scale. Photos: A, C and D: E. Trinkaus; B: M. Samsel.

**Fig 4:**
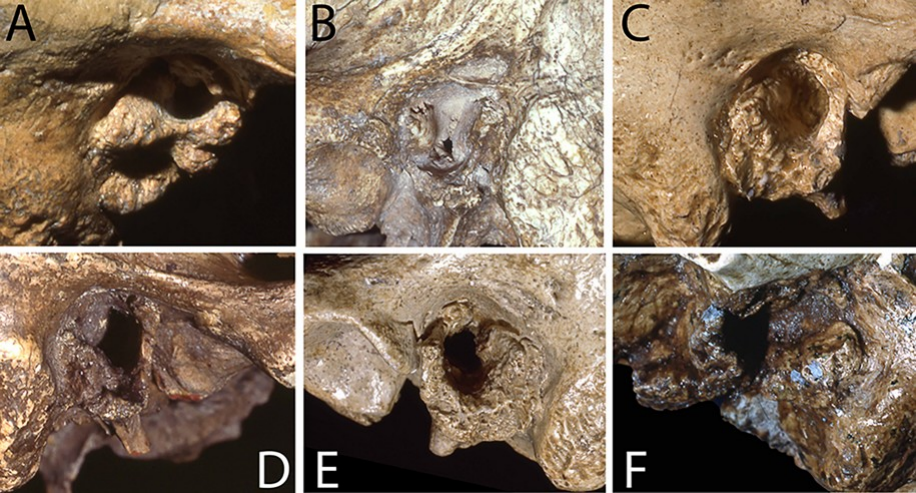
Examples of external auditory exostoses (EAE) among Neandertal specimens. A: Shanidar 1 right (grade 3); B: La Chapelle-aux-Saints 1 left (grade 2); C: Krapina 3 right (grade 1); D: Spy 1 right (grade 2); Krapina 39.1 left (grade 2); Tabun 1 left (grade 2). Not to scale. Photos: A, B, C, E and F: E. Trinkaus; D: H. Rougier.

The presence/absence frequencies for EAE are reflected as well in the distributions of the severity grades across the samples (Fig 2). The EAE for the recent human “dry” and E/MUP samples are restricted to grade 1, larger numbers of individuals (but still a modest number) with grades 2 and 3 are present in the recent “wet” samples. There is also a grade 2 in the LUP sample for one cranium, Iboussières A (Fig 3), and the grade 1 value represents one cranium, Laugerie Basse 4. In contrast, the Neandertal sample includes a specimen with bilateral grade 3 (Shanidar 1 [7]; Fig 4), three with grade 2 on the one sufficiently preserved side (Krapina 39.1, Spy 1 and Tabun 1), and one with grade 2 unilaterally (La Chapelle-aux-Saints 1) (Fig 4). Therefore, 19.6% of the Neandertal sample presents grades 2 or 3, with an additional 37.0% exhibiting grade 1. Among the recent human samples providing grade frequencies, only the Körtik Tepe sample comes close to the Neandertal grade distribution, with a grade 2 plus 3 frequency of 13.5% and a grade 1 frequency of 34.6% (S3 Table).

### Age and sex external auditory exostosis differences

Even though there is little difference across the primary samples in terms of younger versus older individuals, it is possible that some of the more developed Neandertal EAE is partially age-related. The only Late Pleistocene individual with grade 3 exostoses, Shanidar 1, is an older adult based on dental wear and pubic symphyseal morphology [6]. The older La Chapelle-aux-Saints 1, based on its auricular surface [57], exhibits grade 2 EAE on one side, but grade 1 in the other meatus. The other individuals with grade 2 exostoses, Krapina 39.1, Spy 1, Tabun 1 and Iboussières A, are all younger adults, the latter three based on dental wear and the first on the age distribution of the Krapina sample [58].

It is possible to evaluate the degree of sexual difference in EAE presence for the Neandertal and E/MUP samples, despite the small sample sizes per sex; the other samples are too small or have too few EAE cases to provide a reliable assessment. In the Neandertal sample, for the 12 individuals for which sex can be assigned or estimated, EAE occurs in 60.0% of the males (n = 8) and 75% of the females (n = 4). In the 19 sexable E/MUP specimens, EAE are present in 16.7% of males (n = 12) and 28.6% of females (n = 7). The females have modestly higher frequencies in both samples, but in neither case are the sex frequencies significantly different from 0.5 (binomial p = 0.453 and 0.453 respectively). The pattern of higher male frequencies in many recent human samples therefore does not appear to be present among these Late Pleistocene humans.

## Discussion

### Cold water and external auditory exostoses

In her 1986 analysis of global distributions of EAE, Kennedy [18] argued that EAE should be most frequent among middle latitude populations, given the presumed avoidance of cold water at high latitudes and the insufficient stimulation of low latitude warmer water to produce EAE. Subsequent research (Fig 1) has confirmed her hypothesis for high latitude samples, but it has provided a more mixed perspective for middle and low latitudes. Middle latitude samples do generally have higher frequencies of EAE. Yet, in both the middle latitude and equatorial regions, coastal/riverine/lacustrine samples exhibit consistently higher frequencies of EAE overall (Fig 1), and regional studies of archeological samples with more terrestrial versus aquatic resource exploitation provide similar contrasts [19,32–34]. Moreover, it has become evident that cephalic immersion in cold water is not necessary to promote EAE environmentally, and that exposure to cold and damp (maritime) winds is sufficient [31,33].

Therefore, as discussed above (see esp. [10]), there is a strong, but not deterministic, relationship between cold water exposure (especially when associated with wind chill) and the prevalence of EAE (see also [23]). It remains possible that there is an inherited predisposition to developing EAE. It is reflected in differential vasodilation susceptibility of the auditory canal to cold water irrigation, found experimentally in rodents [25] and in the varying degrees of individual EAE development evident in aquatic sport samples subjected to similar environmental conditions [26,28,50,59]. Yet, it is unknown whether there are populational differences in EAE susceptibility, and it appears likely that recent human population differences in EAE are primarily related to environment and behavior.

In this context, the frequency of EAE in the LUP sample is unexceptional relative to recent humans. It is low relative to the middle latitude “wet” samples, and moderately high for the low latitude “dry” and the high latitude samples. Among the sites, Moča, Oberkassel and Zlatý kůn are modestly north of 45° N (all <52° N), and the southern French, northern Italian and Swiss sites distribute around the 45° N latitude. Only the Sicilian site of San Teodoro and the Israeli one of Ohalo II are below 40°. It nonetheless remains unclear how much colder these sites may have been during MIS 2 [60]. The only coastal sites are Arene Candide and San Teodoro, but Ohalo II is on the edge of the Sea of Galilee and Moča 1 was found in Danube River gravels. There is abundant evidence for aquatic resource exploitation at Ohalo II [61], but coastal resources seem not to have been particularly exploited at San Teodoro [62]; it is unclear to what extent marine resources were utilized at Arene Candide (besides shells for beads) [63,64], and no relevant data exist for Moča [65]. The two specimens with EAE, Iboussières A and Laugerie Basse 4, derive from inland sites, although the former site is not far from the Rhône and the latter one overlooks the Vézère. It is therefore difficult to assess whether the low frequency of EAE in the LUP sample is related to the generally higher latitudes of most of the specimens, even though its overall EAE frequency is unexceptional for many recent human samples.

The E/MUP sample provides a higher overall frequency of EAE, albeit all grade 1. The sites yielding observable auditory meatus range from 44° to 56° N, and the five specimens with EAE on at least one side span this geographic range (from Cioclovina 1 at 45° to Sunghir 1 at 56°). Of these specimens, only one (Bausu da Ture 2) can be considered coastal [66], even at MIS 3 lower sea levels, and all of them should have experienced temperatures substantially below the later Holocene ones of the recent human samples [67]. The E/MUP sample should therefore be compared primarily to the recent human high latitude ones; its EAE frequency of 20.8% is slightly below the maximum values of 21.1% and 27.7% for the high latitude “dry” and “wet” groups, but substantially above their means of 3.6% and 3.4% and medians of 0.0% and 2.0%. Even the lower limit of its 95% CI (4.8%) exceeds the means of these recent human groups.

If the E/MUP EAE frequency is moderately high for the relevant recent human environmental group, the Neandertal one is exceptional. As noted, only six of the recent human samples have frequencies above its 95% CI lower limit, and only two “wet” samples provide frequencies similar to the Neandertal one. The Neandertal sample spans middle to high latitudes, from 33° to 50°. Only three of the specimens can be considered as coastal (Forbes’ Quarry 1, Guattari 1 and Tabun 1) [68–70], although Amud 1 was close to the Sea of Galilee [71], and many of the other Neandertals were adjacent to or near streams or modest rivers. They range paleoclimatically from temperate zones in early MIS 5 Europe (Krapina) and MIS 5 to 3 southwest Asia (Amud, Shanidar and Tabun) plus extreme southwestern Europe (Forbes’ Quarry), to substantially colder MIS 3 climates at La Chapelle-aux-Saints, La Ferrassie, La Quina, and Spy (see [60] and [72] for general western Eurasian Late Pleistocene climatic patterns). It is therefore difficult to correlate the high frequency of EAE (including their higher grades of EAE) among these Neandertals with climatic factors.

Evidence for aquatic resource exploitation among these Neandertals varies considerably, in part due to older excavations (and hence partial recovery of small fish bones) at a number of the sites yielding human remains with sufficiently intact temporal bones. There is skeletal and residue analysis evidence for the exploitation of fish among Neandertals in western Europe [73–76], cases of littoral mollusk exploitation along the Mediterranean and coastal Iberia [68,77,78], marine mammal use in southern Iberia [79], and crustacean use in western Iberia [80] (see also [81]). In addition, there are probable water lily starch grains from the Spy Neandertal dental calculus [82]. Yet, stable isotope analysis of 29 Neandertals (albeit all inland, but including the two Spy adults with their EAE) suggests little exploitation of freshwater vertebrate resources [83–85].

Although there is no evidence of littoral resource exploitation in the region of Tabun Cave, there is evidence of mollusk use further north on the eastern Mediterranean coast [77,86]. Therefore, it may be reasonable to attribute the advanced (grade 2) EAE of Tabun 1 to her coastal environment, and it is possible that some of the inland Neandertals with EAE were engaged in freshwater aquatic resource foraging. It nonetheless appears difficult to ascribe their overall high frequency of EAE solely to maritime/riverine/lacustrine environments.

In addition, although most of the EAE present in Neandertal auditory canals are characteristic rounded, knob-like growths (Fig 4; S1 Table), three of the auditory canals that exhibit growths are more irregular in their extra bone formations. These meatus include Amud 1, La Ferrassie 1 left, and La Quina 27. There is also extra posterior canal thickening of the Krapina 3 and 39.1 and the Tabun 1 canals (Fig 4). These variations in the growths raise the question as to whether all of the Neandertal EAE represent responses to cold water irritation of the canal, and whether some of them might be due to alternative etiologies. Yet, even if Amud 1 and La Quina 27 are not included among those with EAE (the La Ferrassie 1 right porus exhibits a more typical EAE growth and hence is scored as having EAE), the Neandertal frequency remains high, at 47.8% (CI: 26.8% - 69.4%).

### Archaic versus Early modern human external auditory exostoses

There is therefore a substantial decrease in EAE frequency from Neandertals to early modern humans in western Eurasia, with a possible decrease from the earlier to later Upper Paleolithic modern humans. The Upper Paleolithic samples are nonetheless not significantly different from each other despite a lower frequency in the LUP sample (see 95% CIs above); the Neandertal and LUP frequencies are significantly different, and the Neandertal and E/MUP frequencies are modestly less distinct (if not significantly different).

The E/MUP sample is significantly higher in latitude than especially the Neandertal one (Wilcoxon p < 0.001), but they do not differ meaningfully in terms of maritime/riverine/lacustrine versus inland contexts (3 of 23 versus 1 of 24; χ^2^ p = 0.238). The LUP sample is intermediate in its latitudinal distribution (χ^2^ p = 0.849 versus the Neandertal sample), it has the highest number of specimens with maritime/riverine/lacustrine contexts (9 of 21), but it also has the lowest EAE frequency. It is therefore possible that factors other than cold water exposure influenced the differences between the Neandertals and the Upper Paleolithic modern human samples. This inference is supported by the apparent increase in aquatic resource exploitation through the Late Pleistocene, of both littoral and riverine/lacustrine resources [77,87–90].

At the same time, the Middle and Late Pleistocene archaic humans from eastern Eurasia also exhibit a high frequency of EAE. The eastern Middle Pleistocene crania provide a frequency equivalent to the Neandertal one (53.3%, n = 15); the addition of the three early Late Pleistocene archaic humans raises the frequency to 68.8% (n = 18) [5]. Yet, the early modern human remains from eastern Eurasia also exhibit an elevated EAE frequency (58.3%, n = 12). Although some of the eastern Eurasian specimens are associated with aquatic contexts, that alone does not appear sufficient to explain their elevated EAE frequency [5].

These data from western and eastern Eurasia therefore suggest that etiological factors in addition to cold water irritation of the auditory canal may have contributed to their elevated frequency of this condition. Could general meatal sanitary conditions be responsible for the additional growths? Could many of these individuals have had a predisposition to develop exostoses in the auditory canal, at higher frequencies than among most recent human populations? Or are the external auditory exostoses indicating a pattern of aquatic resource foraging prior to the Upper Paleolithic that is poorly documented in the Eurasian archeological and stable isotopic records?

## Conclusions

Building on the previously scattered observations of external auditory exostoses among the Neandertals, it appears that there was a very high frequency of EAE among these late archaic humans, yet unexceptional levels of them (relative to most recent human samples) among western Eurasian early modern humans. The frequency of EAE for the E/MUP sample is nonetheless moderately high for a sample that should have been paleoclimatically similar to recent human high latitude (>45°) populations. It remains likely that the high level of EAE among the Neandertals, and the E/MUP samples given its paleoenvironmental context, is due in part to the exploitation of aquatic resources. However, the Neandertal frequency is at the upper limits of recent human population values and is matched only by those who experienced cold water maritime climates. It is therefore likely that, as with eastern Eurasian later archaic humans, multiple factors were involved in their abundance of EAE.

## Supporting information

S1 Table External auditory exostoses (EAE) for western Eurasian late Middle and Late Pleistocene human remains. (PDF)

S2 Table Frequencies of external auditory exostosis (EAE) presence in samples of recent humans. (PDF)

S3 Table Distributions of external auditory exostosis grades among the recent human samples providing grades of severity. (PDF)

S1 File Acknowledgments; supplementary information references. (PDF)

## References

[1] Boule M. L’homme fossile de La Chapelle-aux-Saints. Ann Paléontol. 1911–13; 6:111–72; 7:21–56, 85–192; 8:1–70.

[2] Weidenreich F. The skull of *Sinanthropus pekinensis*. Palaeontol Sinica. 1943; 10D:1–485.

[3] Pérez PJ, Gracia A, Martínez I, Arsuaga JL. Paleopathological evidence of the cranial remains from the Sima de los Huesos Middle Pleistocene site (Sierra de Atapuerca, Spain). Description and preliminary inferences. J Hum Evol. 1997; 33(3):409–21. 9300348 10.1006/jhev.1997.0139

[4] Condemi S. Les Néandertaliens de La Chaise. Paris: Comité des Travaux Historiques et Scientifiques; 2001.

[5] Trinkaus E, Wu XJ. External auditory exostoses in the Xuchang and Xujiayao human remains: Patterns and implications among eastern Eurasian Middle and Late Pleistocene crania. PLoS ONE 2017; 12(12): e0189390 10.1371/journal.pone.0189390 29232394 PMC5726651

[6] Trinkaus E. The Shanidar Neandertals. New York: Academic; 1983.

[7] Trinkaus E, Villotte S. External auditory exostoses and hearing loss in the Shanidar 1 Neandertal. PLoS ONE 2017; 12(10):e0186684 10.1371/journal.pone.0186684 29053746 PMC5650169

[8] Suzuki H. Skulls of the Minatogawa man. Bull Univ Mus Univ Tokyo. 1982; 19:7–49.

[9] Aymard I. Étude Paléopathologique des Vestiges Humains Aziliens de l’Aven des Iboussières (Malataverne, Drôme). Diplôme d’État de Docteur en Médecine, Université de Nantes. Available at: http://www.sudoc.fr/095039856.

[10] Villotte S, Knüsel CJ. External auditory exostoses and prehistoric aquatic resource procurement. J Archaeol Sci Rep. 2016; 6(4):633–6. 10.1016/j.jasrep.2015.05.013

[11] Welcker H. Ueber knöcherne Verengerung und Verschliessung des äusseren Gehörganges. Archiv Ohrenheilkunde 1864; 1:163–74.

[12] Jackson G. The etiology of exostoses of the external auditory meatus. Br Med J. 1909; 2(2546):1137–8.

[13] Hrdlička A. Ear exostoses. Smithson Misc Coll. 1935; 93(6):1–100.

[14] Snow CE. Indian Knoll Skeletons of Site Oh 2, Ohio County, Kentucky. Univ Kentucky Rep Anthropol. 1948; 4(3-II):371–554.

[15] Berry A, Berry R. Epigenetic variation in the human cranium. J Anat. 1967; 101:361–79. 4227311 PMC1270890

[16] Hauser G, DeStefano CF. Epigenetic Variants of the Human Skull. Stuttgart: Schweizerbart’sche; 1989.

[17] Hanihara T, Ishida H. Frequency variations of discrete cranial traits in major human populations. III. Hyperostotic variations. J Anat. 2001; 199:251–72. 10.1046/j.1469-7580.2001.19930251.x 11554504 PMC1468329

[18] Kennedy GE. The relationship between auditory exostoses and cold water: a latitudinal analysis. Am J Phys Anthropol. 1986; 71(4):401–15. 10.1002/ajpa.1330710403 3812656

[19] Standen VG, Arriaza B. Santoro CM. External auditory exostosis in prehistoric Chilean populations: a test of the cold water hypothesis. Am J Phys Anthropol. 1997; 103(1):119–29. 10.1002/(SICI)1096-8644(199705)103:1<119::AID-AJPA8>3.0.CO;2-R 9185955

[20] Ponce P, Ghidini G, González-José R. External auditory exostosis “at the end of the world”: the southernmost evidence according to the latitudinal hypothesis. Brit Archaeol Rep. 2008; S1743:101–7.

[21] Kuzminsky SC, Erlandson JM, Xifara T. External auditory exostoses and its relationship to prehistoric abalone harvesting on Santa Rosa Island, California. Intl J Osteoarchaeol. 2016; 26:1014–23. 10.1002/oa.2512

[22] Leonetti JP, Marzo SJ. Diseases of the external auditory canal. In: Pensak ML, Choo DI, editors. Clinical Otology, 4th ed New York: Thieme; 2015 p. 181–91.

[23] Van Gilse PHG. Des observations ulterieures sur la genèse des exostoses du conduit externe par l’iritation d’eau froide. Acta Oto-laryngol. 1938; 26:343–52.

[24] Harrison DFN. Exostosis of the external auditory meatus. J Laryngol Otol. 1951; 65:704–14. 14880790 10.1017/s0022215100010641

[25] Harrison DFN. The relationship of osteomata of the external auditory meatus to swimming. Ann Roy Coll Surg. 1962; 31(9):187–201. PMC2414240 13904891

[26] Cooper A, Tong R, Neil R, Owens D, Tomkinson A. External auditory canal exostoses in white water kayakers. Br J Sports Med. 2010; 44:144–7. 10.1136/bjsm.2008.048157 18603582

[27] Ito M, Ikeda M. Does cold water truly promote diver’s ear? Undersea Hyperbaric Med. 1998; 25(1):59–62. 9566088

[28] Kroon DF, Lawson ML, Derkay CS, Hoffmann K, McCook J. Surfer’s ear: External auditory exostoses are more prevalent in cold water surfers. Otolaryngol Head Neck Surg. 2002; 126(5):499–504. 10.1067/mhn.2002.124474 12075223

[29] Altuna X, Gómez J, Luqui I, Vea JC, Algaba J. Prevalence of exostoses among surfers of the Basque coast. Acta Otorrinolaringol Esp. 2004; 55:364–68. 15552211 10.1016/s0001-6519(04)78537-4

[30] Hurst W, Bailey M, Hurst B. Prevalence of external auditory canal exostoses in Australian surfboard riders. J Laryngol Otol 2004; 118:348–31. 10.1258/002221504323086525 15165308

[31] King JF, Kinney AC, Iacobellis SF, Alexander TH, Harris JP, Torre P, et al Laterality of exostosis in surfers due to evaporative cooling effect. Otol Neurotol 2010; 31(2):345–51. 10.1097/MAO.0b013e3181be6b2d 19806064

[32] Villotte S, Stefanović S, Knüsel CJ. External auditory exostoses and aquatic activities during the Mesolithic and the Neolithic in Europe: results from a large prehistoric sample. Anthropol (Brno). 2014; 52(1):73–89.

[33] Okamura MMM, Boyadjian CHC, Eggers S. Auditory exostoses as an aquatic activity marker: A comparison of coastal and inland skeletal remains from tropical and subtropical regions of Brazil. Am J Phys Anthropol. 2007; 132:558–67. 10.1002/ajpa.20544 17243122

[34] Arnay-de-la-Rosa M, González-Reimers E, Velasco-Vázquez J, Santolaria-Fernández F. Auricular exostoses among the prehistoric population of different islands of the Canary archipelago. Ann Otol Rhinol Laryngol. 2001; 110(11):1080–3. 10.1177/000348940111001117 11713923

[35] Timofeev I, Notkina N, Smith IM. Exostoses of the external auditory canal: a long-term follow-up study of surgical treatment. Clin Otolaryngol. 2004; 29:588–94. 10.1111/j.1365-2273.2004.00865.x 15533142

[36] Filipo R, Fabiani M, Barbara M. External ear canal exostosis: a physiopathological lesion in aquatic sports. J Sports Med. 1982; 22:329–36. 7162188

[37] Rabach LA, Kveton JF. Clinical evaluation of hearing loss. In: Pensak ML, Choo DI, editors. Clinical Otology, 4th ed New York: Thieme; 2015 p. 139–47.

[38] White RD, Ananthakrishnan G, McKean SA, Brunton JN, Hussain SSM, Sudarshan TA. Masses and disease entities of the external auditory canal: radiological and clinical correlation. Clin Radiol. 2011; 67(2):172–81. 10.1016/j.crad.2011.08.019 22018812

[39] Guest JF, Greener MJ, Robinson AC, Smith AF. Impacted cerumen: composition, production, epidemiology and management. Quart J Med. 2004; 97(8):477–88. 10.1093/qjmed/hch082 15256605

[40] Gregg JB, McGrew RN. Hrdlička revisited (external auditory canal exostoses). Am J Phys Anthropol. 1970; 33:37–40. 10.1002/ajpa.1330330106 5431485

[41] Crowe F, Sperduti A, O’Connell TC, Craig OE, Kirsanow K, Germoni P, et al Water-related occupations and diet in two Roman coastal communities (Italy, first to third century AD): correlation between stable carbon and nitrogen isotope values and auricular exostosis prevalence. Am J Phys Anthropol. 2010; 142(3):355–66. 10.1002/ajpa.21229 20014179

[42] Ponzetta MT, Hauser G, Vienna A. Auditory hyperostosis and the environment: an update. Int J Anthropol. 1997; 12(2):29–42.

[43] Mazza B. Auditory exostoses in Pre-Hispanic populations of the Lower Paraná wetlands, Argentina. Int J Osteoarchaeol. 2016; 26:420–30. 10.1002/oa.2432

[44] Roche AF. Aural exostoses in Australian aboriginal skulls. Ann Otol Rhinol Laryngol 1964; 73:82–91. 10.1177/000348946407300109 14128721

[45] Frayer DW. Auditory exostoses and evidence for fishing at Vlasac. Curr Anthropol. 1988; 29(2):346–9.

[46] Graham MD. Osteomas and exostoses of the external auditory canal. A clinical, histopathologic and scanning electron microscopic study. Ann Otol Rhinol Laryngol 1979; 88: 566–72. 10.1177/000348947908800422 475257

[47] Velemínská J, Brůžek J. Early Modern Humans from Předmostí near Přerov, Czech Republic. A New Reading of Old Documentation. Prague: Academia; 2008.

[48] Trinkaus E. Late Pleistocene adult mortality patterns and modern human establishment. Proc Natl Acad Sci USA. 2011; 108:1267–71 10.1073/pnas.1018700108 21220336 PMC3029716

[49] Wong BJF, Cervantes W, Doyle KJ, Karamzadeh AM, Boys P, Brauel G, et al Prevalence of external auditory canal exostoses in surfers. Arch Otolaryngol Head Neck Surg 1999; 125:969–72. 10.1001/archotol.125.9.969 10488981

[50] Nakanishi H, Tono T, Kawano H. Incidence of external auditory canal exostoses in competitive surfers in Japan. Otolaryngol Head Neck Surg 145(1):80–5. 10.1177/0194599811402041 21493286

[51] Koruyucu MM, Şahin FS, Delibaş D, Erdal ÖD, Benz M, Özkaya V. Auditory exostosis: Exploring the daily life at an early sedentary population (Körtik Tepe, Turkey). Intl J Osteoarchaeol. 2018; 28:615–25. 10.1002/oa.2674

[52] Manzi G, Sperduti A, Passarello P. Behavior-induced auditory exostoses in Imperial Roman society: Evidence from coeval urban and rural communities near Rome. Am J Phys Anthropol. 1991; 85:253–60. 10.1002/ajpa.1330850303 1897597

[53] Mehta C, Patel N. StatXact 4 for Windows. Cambridge MA: Cytel Software Corp; 1999.

[54] NCSS 11.0.19. Kaysville, UT: NCSS; 2018. Available from: https://ncss.com/software/ncss.

[55] Soper D. Binomial probability confidence internal calculator. Free Statistics Calculators v. 4.0. https://www.danielsoper.com/statcalc/calculator.aspx?id=85; 2019.

[56] Dutour O, Onrubia-Pintado J. Interactions homme-environnement océanique pendant la préhistoire récente des Iles Canaries: nouvelles données paléoanthropologiques de la région de Galdar (Grande Canarie). C R Acad Sci Paris Série III, 1991; 313:125–30.

[57] Haeusler M, Trinkaus E, Fornai C, Müller J, Bonneau N, Boeni T, et al Morphology, pathology and the vertebral posture of the La Chapelle-aux-Saints Neandertal. Proc Natl Acad Sci USA. 2019; 10.1073/pnas.1820745116 PMC6421410 30804177

[58] Wolpoff MH. The Krapina dental remains. Am J Phys Anthropol. 1979; 50:67–114. 10.1002/ajpa.1330500110 736116

[59] Karegeannes JC. Incidence of bony outgrowths of the external ear canal in U.S. Navy divers. Undersea Hyperbaric Med. 1995; 22(3):301–6. . 7580769

[60] Fletcher WJ, Sánchez-Goñi MF, Allen JRM, Cheddadi R, Combourieu-Nebout N, Huntley B et al Millennial-scale variability during the last glacial in vegetation records from Europe. Quatern Sci Rev. 2010; 29:2839–64. 10.1016/j.quascirev.2009.11.015

[61] Nadel D (ed). Ohalo II–A 23,000-Year-Old Fisher-Hunter-Gatherer’ Camp on the Shore of the Sea of Galilee. Haifa: Hect Museum, Univ of Haifa; 2002.

[62] Mannino M, Di Salvo R, Schimmenti V, Di Patti C, Incarbona A, Sineo L, et al Upper Palaeolithic hunter-gatherer subsistence in Mediterranean coastal environments: an isotopic study of the diets of the earliest directly-dated humans from Sicily. J Archaeol Sci. 2011; 38:3094–100. 10.1016/j.jas.2011.07.009

[63] Bietti A. The Late Upper Paleolithic in Italy: An overview. J World Prehist. 1990; 4:95–155.

[64] Formicola V, Pettitt PB, Maggi R, Hedges R. Tempo and mode of formation of the Late Epigravettian necropolis of Arene Candide cave (Italy): direct radiocarbon evidence. J Archaeol Sci. 2005; 32:1598–1602. 10.1016/j.jas.2005.04.013

[65] Šefčáková A, Katina S, Mizera I, Halouzka R, Barta P, Thurzo M. A late Upper Palaeolithic skull from Moča (the Slovak Republic) in the context of central Europe. Acta Musei Nationalis Pragae. 2011; 67B(1–2):3–24.

[66] Villotte S, Samsel M, Sparacello V. The paleobiology of two adult skeletons from Baousso da Torre (Bausu da Ture) (Liguria, Italy): implications for Gravettian lifestyle. C R Palevol. 2017; 16:462–73. 10.1016/j.crpv.2016.09.004

[67] Fleitmann D, Cheng H, Badertscher S, Edwards RL, Mudelsee M, Göktürk OM, et al Timing and climatic impact of Greenland interstadials recorded in stalagmites from northern Turkey. Geophys Res Lett. 2009; 36: L19707 10.1029/2009GL040050

[68] Fa DA, Finlayson JC, Finlayson G, Giles-Pacheco F, Rodríguez-Vidal J, Gutiérrez-López JM. Marine mollusc exploitation as evidenced by the Gorham’s Cave (Gibraltar) excavations 1998–2005: The Middle-Upper Palaeolithic transition. Quatern Int. 2016; 407:16–28. 10.1016/j.quaint.2015.11.148

[69] Jelinek AJ. The Tabun Cave and Paleolithic man in the Levant. Science. 1982; 25:1369–75. 10.1126/science.216.4553.1369 17798344

[70] Blanc A. The fossil man of Circe’s Mountain. Nat Hist. 1940; 45:280–7.

[71] Suzuki H, Takai F (eds). The Amud Man and his Cave Site. Tokyo: Academic Press of Japan; 1970.

[72] Allen JRM, Hickler T, Singarayer JS, Sykes MT, Valdes PJ, Huntley B. Last glacial vegetation of northern Eurasia. Quatern Sci Rev. 2010; 29:2604–18. 10.1016/j.quascirev.2010.05.031

[73] Le Gall O. Poissons et pêches au Paléolithique (quelques données de l’Europe occidentale). L’Anthropol. 1992; 96(1):121–34.

[74] Muñoz M, Casadevall M. Fish remains from Arbreda Cave (Serinyà, Girona), northeast Spain, and their palaeoecological significance. J Quatern Sci. 1997; 12(2):111–5.

[75] Hardy BL, Moncel MH. Neanderthal use of fish, mammals, birds, starchy plants and wood 125,000–250,000 years ago. PLoS ONE. 2011; 6:e23768 10.1371/journal.pone.0023768 21887315 PMC3161061

[76] Rosello E. La ictiofauna musteriense de Cueva Millán (Burgos): Consideraciónes de indole biologica y cultural contrastadas con ictiocenosis Paleoliticas Cantábricas. Estudios geol. 1992; 48:79–83.

[77] Colonese AC, Mannino MA, Bar-Yosef Mayer DE, Fa DA, Finlayson JC, Lubell D, et al Marine mollusc exploitation in Mediterranean prehistory: An overview. Quatern Int. 2011; 239:86–103. 10.1016/j.quaint.2010.09.001

[78] Gutiérrez-Zugasti I, Rios-Garaizar J, Marín-Arroyo AB, Rasines de Río P, Maroto J, Jones JR, et al A chrono-cultural reassessment of the levels VI-XIV from El Cuco rock-shelter: A new sequence for the Late Middle Paleolithic in the Cantabrian region (northern Iberia). Quatern Intl. 2018; 474:44–55. 10.1016/j.quaint.2017.06.059

[79] Stringer CB, Finlayson JC, Barton RNE, Fernández-Jalvo Y, Cáceres I, Sabin RC, et al 2008 Neanderthal exploitation of marine mammals in Gibraltar. Proc Natl Acad Sci USA. 2008; 105(38):14319–24. 10.1073/pnas.0805474105 18809913 PMC2567146

[80] Zilhão J. Neandertals from the world’s end: results of recent research. In: Turbón D, Fañanás I, Rissech C, Rosa A, editors. Biodiversidad Humana y Evolución Barcelona: Universidad de Barcelona/Sociedad Española de Antropologia Fisica; 2012, p. 68–77.

[81] Álvarez-Fernández E. L’exploitation des ressources marines au Paléolithique moyen et supérieur initial en Europe: synthèse des données disponibles. Palethnologie. 2015; 7 10.4000/palethnologie.

[82] Henry AG, Brooks AS, Piperno DR. Microfossils in calculus demonstrate consumption of plants and cooked foods in Neanderthal diets (Shanidar III, Iraq; Spy I and II, Belgium). Proc Natl Acad Sci USA. 2011; 108:486–91. 10.1073/pnas.1016868108 21187393 PMC3021051

[83] Richards MP, Pettitt PB, Stiner MC, Trinkaus E. Stable isotope evidence for increasing dietary breadth in the European mid-Upper Paleolithic. Proc Natl Acad Sci USA. 2001; 98:6528–32. 10.1073/pnas.111155298 11371652 PMC33502

[84] Wissing C, Rougier H, Crevecoeur I, Germonpré M, Naito Y, Semal P, et al Isotopic evidence for dietary ecology of late Neandertals in north-western Europe. Quatern Int. 2016; 411:327–45. 10.1016/j.quaint.2015.09.091

[85] Jaouen K, Richards MP, Le Cabec A, Welker F, Rendu W, Hublin JJ, et al Exceptionally high δ^14^N values in collagen single amino acids confirm Neandertals as high-trophic level carnivores. Proc Natl Acad Sci USA. 2019; 116:4928–33. 10.1073/pnas.1814087116 30782806 PMC6421459

[86] Stiner M. Prey choice, site occupation intensity & economic diversity in the Middle–early Upper Palaeolithic at the Üçağizli Caves, Turkey. Before Farming. 2009; 2009(3):1–20. 10.3828/bfarm.2009.3.3

[87] Richards MP. Stable isotope evidence for European Upper Paleolithic human diets In: Hublin JJ, Richards MP, editors. The Evolution of Hominin Diets. Dordrecht: Springer; 2009, p. 251–7. 10.1007/978-1-4020-9699-0_20

[88] Straus LG. Iberia Before the Iberians. Albuquerque: University of New Mexico Press; 1992.

[89] Holt B, Formicola V. Hunters of the Ice Age: The biology of Upper Paleolithic people. Yrbk Phys Anthropol. 2008; 51:70–90. 10.1002/ajpa.20950 19003886

[90] Adán GE, Álvarez-Lao D, Turrero P, Arbizu M, García-Vázquez E. Fish as a resource in North Spain during the Upper Paleolithic. J Archaeol Sci. 2009; 36:895–9. 10.1016/j.jas.2008.11.017

