# Supplementary material for: External auditory exostoses among western Eurasian late Middle and Late Pleistocene humans
Source: PLoS One. 2019 Aug 14;14(8):e0220464. doi: 10.1371/journal.pone.0220464 (PMC6693685; doi:10.1371/journal.pone.0220464)
Supplement: S1 Table — (PDF) [file pone.0220464.s001.pdf]

External auditory exostoses among western Eurasian late Middle and Late Pleistocene humans

Supplementary Information

Erik Trinkaus,[1] Mathilde Samsel,[2] and Sébastien Villotte[3]

[1] Department of Anthropology, Washington University, Saint Louis MO 63130, USA. [2] UMR5199 PACEA, Université de Bordeaux, Bâtiment B8, Allée Geoffroy Saint Hilaire CS 50023, 33615 Pessac, France. [3] CNRS, UMR5199 PACEA, Bâtiment B8, Allée Geoffroy Saint Hilaire, 33615 Pessac, France

S1 Later Pleistocene and Recent Human Auditory Exostoses

Table S1. External auditory exostoses (EAE) for western Eurasian late Middle and Late Pleistocene human remains.

	Lat	*Side*	*Age*[1]	*Sex*[2]	*EAM exostoses*	*Grade*	*Source*[3]	*Scorer*[4]	*Ref*[5]
**Late Middle Pleistocene**									
Biache 1	50°	lt	YA	--	absent, thick tympanic with external growths	0	original [1]	[1]	[1]
Biache 2	50°	lt	A	--	absent, thick tympanic	0	cast	ET	[2]
La Chaise-BD 7	46°	rt	A	--	posterior and inferior growths on tympanic	1	original [3]	[3], ET	[3]
Ehringsdorf 9	51°	lt	A	--	absent, very thick tympanic posteroinferior	0	photo [4]	ET	[4]
Reilingen 1	49°	rt	A	--	absent, moderately thick tympanic walls	0	cast	ET	[5]
**Neandertals**									
Amud 1	33°	rt	YA	M	small posterior growth (inferior tympanic missing)	1	cast; photo	ET	[6]
		lt			large filling posterior with internal thickening and additional bone	1			
La Chapelle-aux-Saints 1[6]	45°	rt	OA	M	medium knob anterior, lateral knob posterior	1	original [8]; photo; CT scan	ET	[7,8]
		lt			medium posterior and large anterolateral growths; closed down to very small canal medially	2			
La Ferrassie 1	45°	rt	OA	M	small-to-medium growth mid-posterior internally; small knob anteroinferior	1	[9]; photos	ET	[9]
		lt			spicules and growths in from tympanic around anterior and inferior porus	1			

Table S1 (cont.)

	Lat	*Side*	*Age*[1]	*Sex*[2]	*EAM exostoses*	*Grade*	*Source*[3]	*Scorer*[4]	*Ref*[5]
La Ferrassie 2	45°	rt	OA	F	absent	0	photos	ET	[9]
Forbes' Quarry 1	36°	rt	OA	--	irregular margins but no apparent growths	0	photos	ET	[10]
Guattari 1	41°	rt	OA	(M)	very small superior and anteroinferior growths, otherwise round	1	photos	ET	[11]
		lt			small superior and small posterior growths, interior rounded ones	1			
Krapina 3[7]	46°	rt	Adol/YA	(F)	distinct rounded posterior middle protrusion	1	photo; cast	ET	[12,13]
Krapina 5	46°	rt	Adol/YA	(M)	absent	0	cast	ET	[12,13]
Krapina 10	46°	lt	Adol/YA	--	absent	0	photo	ET	[12,13]
Krapina 38.1	46°	rt	Adol/YA	--	absent, thick tympanic	0	photo	ET	[12,13]
Krapina 38.7	46°	rt	Adol/YA	--	absent, thick tympanic, possible posterosuperior new bone	0	photo	ET	[12,13]
Krapina 38.16	46°	rt	Adol/YA	--	medium posterosuperior knob	1	photo	ET	[12,13]
Krapina 39.1	46°	lt	Adol/YA	--	medium anterosuperior growth; large knob posteriorly on thick tympanic	2	original [3]; photo	[3], ET	[12,13]
La Quina 5	45°	rt	YA	--	absent	0	original [14]; photo; cast	[14], ET	[14]
		lt			absent	0			
La Quina 10	45°	lt	A	--	absent, thickening of tympanic anterior and posterior	0	photo	ET	[14]
La Quina 27	45°	rt	A	--	broad irregular growth across inferior porus from anteroinferior to posterior, superoposterior spicules, thick tympanic	1	photos	ET	[14]
Saccopastore 1	42°	lt	YA	(F)	small knob from posteroinferior tympanic	1	photos	ET	[15]
Saccopastore 2	42°	rt	YA	(M)	absent, thick tympanic	0	photos	ET	[15]
Shanidar 1[6]	37°	rt	OA	M	large and bridging exostosis across porus	3	original; [16,17]; photos	ET	[17]
		lt			large posterior growth, superior and inferior internal knobs	3			
Shanidar 5	37°	lt	OA	(M)	absent, thick tympanic	0	original; cast; photos	ET	[17]
Spy 1 (I)	50°	rt	YA	--	large posterior superior and inferior growths	2	photo	ET	[18,19]

Table S1 (cont.)

	Lat	*Side*	*Age*[1]	*Sex*[2]	*EAM exostoses*	*Grade*	*Source*[3]	*Scorer*[4]	*Ref*[5]
Spy 10 (II)	50°	rt	YA	--	modest posterior interior knob in broad posterior swelling	1	photos	ET	[18,19]
		lt			small posterior knob with posteroinferior thick tympanic	1			
Tabun 1	33°	rt	YA	F	small rounded posterior growth, large anterosuperior growth	2	photos	ET, SV	[20,21]
**Middle Paleolithic Modern Humans**									
Qafzeh 6	33°	rt	YA	--	absent	0	cast; photo	ET	[22,23]
		lt			thin growth along posterosuperior porus	0			
Qafzeh 9	33°	rt	Adol	F	absent	0	cast; photo	ET	[22,23]
Skhul 5	33°	rt	YA	M	absent (anterior tympanic missing)	0	cast; photo	ET	[20,24]
		lt			absent	0			
Skhul 6	33°	lt	YA	--	growths inferiorly and around posterior tympanic	1	photo	ET, SV	[20,24]
**Early/Mid Upper Paleolithic**									
Bausu da Ture 2	44°	lt	YA	M	absent	0	original [25]	SV	[25]
Brno 2	49°	lt	OA	(M)	absent, thick tympanic	0	photo	ET	[26,27]
Cioclovina 1	45°	rt	YA	(M)	clear rounded posterosuperior protrusion	1	photos	ET	[28,29]
		lt			clear rounded swelling along posterior canal	1			
Cro-Magnon 1	45°	rt	OA	M	absent	0	original	SV, ET	[30-32]
Cro-Magnon 2	45°	lt	OA	(F)	absent	0	original	SV, ET	[30-32]
Dolní Věstonice 3	49°	rt	YA	F	absent	0	photos	ET	[33]
		lt			absent	0			
Dolní Věstonice 13	49°	rt	YA	M	absent, thick tympanic	0	original	ET	[33]
		lt			absent, thick tympanic	0			
Dolní Věstonice 14	49°	lt	Adol	M	absent	0	original	ET	[33]
Dolní Věstonice 15	49°	rt	YA	M	absent	0	original	ET	[33]
		lt			absent	0			
Dolní Věstonice 16	49°	rt	OA	M	absent	0	original	ET	[33]
		lt			absent	0			

Table S1 (cont.)

	Lat	*Side*	*Age*[1]	*Sex*[2]	*EAM exostoses*	*Grade*	*Source*[3]	*Scorer*[4]	*Ref*[5]
Mladeč 1	50°	lt	Adol	(F)	absent	0	photo	ET	[34]
Mladeč 2	50°	lt	Adol	(F)	inferior, inferoposterior and superoposterior protruding bony spicules	1	photo	ET	[34]
Mladeč 5	50°	rt	A	(M)	absent	0	photo	ET	[34]
Muierii 2	45°	lt	A	--	absent	0	original	ET	[35]
Oase 2	45°	lt	Adol	--	small rounded posterior growth, beginning of a small anteroinferior growth	1	original	ET	[36]
Pataud 1	45°	rt	YA	F	absent	0	original	SV, ET	[37]
		lt			tiny knob posteriorly within canal	0			
Pavlov 1	49°	rt	OA	(M)	very small swelling inferoanterior	0	original	ET	[33]
		lt			absent	0			
Předmostí 1	49°	rt	Adol	--	absent	0	photo [39]	ET, SV	[38,39]
Předmostí 4	49°	rt	YA	F	inferior growths closing one-third of porus	1	photo [39]	ET, SV	[38,39]
Předmostí 9	49°	rt	YA	M	absent	0	photo [39]	ET, SV	[38,39]
Předmostí 10	49°	rt	YA	F	small posterosuperior growths	0	photo [39]	ET, SV	[38,39]
Sunghir 1	56°	rt	OA	M	small irregular growths anteroinferior; swelling with fine protrusions mid-posterior, closing of the inferior porus	1	original; photos	ET	[40]
		lt			absent	0			
Sunghir 5	56°	lt	A	--	absent	0	photo	ET	[40]
Vilhonneur 1	46°	rt	Adol	--	absent	0	photo [41]	ET	[41]
**Late Upper Paleolithic**									
Arene Candide 2	44°	rt	YA	M	absent	0	scan [42]	VS	[43]
		lt			absent	0			
Arene Candide 4	44°	lt	YA	M	absent	0	scan [44]	VS	[43]
Arene Candide 5	44°	rt	YA	M	absent	0	scan [45]	VS	[43]
Arene Candide 12	44°	rt	A	M	absent	0	scan [46]	VS	[43]
		lt				0			
Arene Candide 16	44°	rt	Adol	M	absent	0	scan [47]	VS	[43]
		lt			absent	0			

Table S1 (cont.)

	Lat	*Side*	*Age*[1]	*Sex*[2]	*EAM exostoses*	*Grade*	*Source*[3]	*Scorer*[4]	*Ref*[5]
Bichon 1	47°	rt	YA	M	absent	0	original	MS	[48]
		lt			absent	0			
Chancelade 1	45°	rt	OA	(F)	absent	0	original	MS	[49,50]
		lt			absent	0			
Iboussières A[6]	44°	rt	YA	M	large rounded growth mid-posterior, with smaller swelling medial of it and extending to mid-inferior; low swelling along anterior tympanic	2	original; [51]	MS, SV	[52,53]
		lt			bilobular growths mid-posterior and posteroinferior, broad swelling anteroinferior, and small swelling posterosuperior	2			
Lafaye (Bruniquel) 1	44°	rt	YA	F	absent	0	original	MS	[54,55]
		lt			absent	0			
Laugerie Basse 4	45°	rt	OA	(M)	small swelling along mid-posterior porus	1	original	MS	[55,56]
		lt			diffuse growth along inferior meatus	1			
Moča 1	48°	rt	OA	(F)	tiny smooth swelling along superior tympanic margin	0	[57]; photos	ET, SV	[57]
		lt			tiny smooth swelling along superior tympanic margin	0			
Oberkassel 1	51°	rt	OA	M	absent	0	CT scan	ET	[58]
		lt			absent	0			
Oberkassel 2	51°	rt	YA	F	absent	0	CT scan	ET	[58]
		lt			absent	0			
Ohalo 2	33°	lt	OA	M	absent	0	cast	ET	[59,60]
Le Peyrat 5	45°	rt	A	M	absent	0	original	MS	[61,62]
Rochereil 1	45°	rt	OA	--	absent	0	original	MS	[63]
		lt			absent	0			
St Germain-la-Rivière 4	45°	rt	YA	F	absent	0	original	MS	[64]
		lt			absent	0			
San Teodoro 1	38°	lt	YA	F	absent, small lateral tympanic nubbins	0	scan; original	MS, VS	[65,66]

Table S1 (cont.)

	Lat	*Side*	*Age*[1]	*Sex*[2]	*EAM exostoses*	*Grade*	*Source*[3]	*Scorer*[4]	*Ref*[5]
San Teodoro 2	38°	rt	A	--	absent	0	original;	MS, VS	[65,66]
		lt			absent	0	scan		
Villabruna 1	46°	lt	YA	M	absent	0	scan; photo	VS, ET	[67]
Zlatý kůň 1	50°	rt	A	(F)	absent	0	photo [19]	ET	[68]

[1] The age categories are approximate, and the crania are divided into later adolescents (Adol), younger adults less than ≈40 years (YA) and older adults greater than ≈40 years. The adolescents are all ≥15 years and are considered adolescent based largely on the incomplete eruption of the M3s. A number of them (e.g., Dolní Věstonice 14, Mladeč 1 and 2) are routinely considered as though they are mature, and it is unclear whether the Krapina remains are late adolescent or young adult (see note 7).

[2] Sex indications without parentheses are based on pelvic remains. Those designations placed within parentheses are based on combinations of craniofacial rugosity and body size, as available. The latter designations are less certain for the Neandertals in particular, although those for individuals from the sites of Krapina, Saccopastore and Shanidar are based on ranges of variation with each site sample.

[3] The source indicates the previous publication of EAE presence/absence for the specimen (for the few that have been published), and whether the observations were made by one of us on the original fossil, a high quality (resin) cast of the fossil, high quality photograph(s), CT scans, and/or digital surface scans. "Original" followed by a reference indicates an observation on the original specimen by the author of the publication. For photographic scoring, the reference is provided if a published photograph was employed; otherwise the photograph(s) are by the authors. In some cases (e.g., the Shanidar and Sunghir remains), the original observations on EAE were made on the original specimens, but the grade scoring was done from photographs and/or casts.

[4] The initials of the author(s) primarily scoring the grade of EAE are provided, and all cases were double checked by the others. In cases of published EAE observations, the original comments have been translated into grade scores, resulting in a reference and an author's initials. MS: M. Samsel; ET: E. Trinkaus; SV: S. Villotte; VS: personal communication from V.S. Sparacello.

[5] Reference providing basic context and description of the skeletal remains.

[6] The La Chapelle-aux-Saints 1, Shanidar 1 and Iboussières A partial skeletons each exhibits a suite of pathological lesions, including trauma, enthesopathies, and osteoarthritis [17,51,69,70]. Although their bilateral EAE were noted and briefly described (but not given grades) by Boule [7], Trinkaus [17] and Aymard [51] respectively (but see [71] for Shanidar 1), none of their EAE appear to be related to the other lesions on the remains, most of which can be attributed to the rigors of a Pleistocene foraging existence. Only the DISH of Shanidar 1 [72] and the femoral diaphyseal lesions of Iboussières A [51] are unusual for a Pleistocene human, and they are unlikely to have affected their auditory canals.

[7] None of the included Krapina crania (Krapina 3, 5 and 10) or isolated temporal bones (Krapina 38.# and 39.1) preserve reliable adolescent versus adult or younger versus older adult age indicators. However, all of these temporal bones appear fully mature [73] and the associated dental remains provide an age range from juvenile to young adult [74]. These Krapina specimens are therefore considered as adolescent/young adult.
