# Supplementary material for: External auditory exostoses among western Eurasian late Middle and Late Pleistocene humans
Source: PLoS One. 2019 Aug 14;14(8):e0220464. doi: 10.1371/journal.pone.0220464 (PMC6693685; doi:10.1371/journal.pone.0220464)
Supplement: S2 Table — (PDF) [file pone.0220464.s002.pdf]

External auditory exostoses among western Eurasian late Middle and Late Pleistocene humans

Supplementary Information

Erik Trinkaus,[1] Mathilde Samsel,[2] and Sébastien Villotte[3]

[1] Department of Anthropology, Washington University, Saint Louis MO 63130, USA. [2] UMR5199 PACEA, Université de Bordeaux, Bâtiment B8, Allée Geoffroy Saint Hilaire CS 50023, 33615 Pessac, France. [3] CNRS, UMR5199 PACEA, Bâtiment B8, Allée Geoffroy Saint Hilaire, 33615 Pessac, France

S1 Later Pleistocene and Recent Human Auditory Exostoses

Table S2. Frequencies (%) of external auditory exostosis (EAE) presence in samples of recent humans.

The samples are grouped into low (<30°), middle (30° - 45°) and high (>45°) latitude samples following Kennedy [75], and within each, into "wet" (coastal/riverine/lacustrine) and "dry" (inland with little or no aquatic exploitation).

The samples only include adults and later adolescents (>≈15 years). Male and female samples are combined, given that the data are not available by sex for a substantial number of the recent human samples. It is nonetheless recognized that a number of studies (e.g., [76-81]) have found substantial differences in the male versus female frequencies, with the males often having the higher incidence. Small (n < 30) samples are not included.

In the allocation of the samples into the "wet" versus "dry" categories, several factors were employed. In cases in which the authors have provided explicit contrasts between coastal/riverine versus terrestrial residence/occupation/resource exploitation (e.g., [78-80,82-84]), their divisions have been followed. For inland localities with little or no evidence of aquatic resource exploitation, the samples have been considered "dry." Yet, inland (riverine) sites are considered "wet" where there is evidence of aquatic resource exploitation (e.g., Iron Gates Mesolithic sites, Indian Knoll). Coastal samples, even if there is no associated evidence for the exploitation of littoral resources, have been placed in the "wet" category, given the effects of general maritime exposure and associated wind chill [80,85]. Pooled samples based on national boundaries, immigrant groups, and pooled samples from very large islands with both coastal and inland areas are not included. It is fully recognized that some of the samples considered to be "wet" or "dry" could be placed in the other category. However, such resorting is not likely to substantially alter the "wet"/"dry" distributions within each of the latitudinal zones.

It is also recognized that the latitudinal zones, with cut-offs at 30° and 45°, are partially arbitrary, especially given variation in sea temperatures at a given latitude due to major oceanic currents. Those samples which fall close to a latitudinal boundary (e.g., Chinook, Arikara) have been placed in the higher latitude sample. These comments only serve to reinforce that such a global analysis, in contrast to detailed ones within regions, primarily serve to highlight overall patterns. They are employed here, not to resolve the detailed patterns of EAE incidence across recent human samples, but to provide a framework for evaluating the western Eurasian later Pleistocene distributions.

Site	*Frequency (%)*	*n*	*Reference*
**Low Latitude (<30°) "dry"**			n = 22
Australia-North	1.5	172	[75]
Australia-Central	3.2	127	[75]

Site	*Frequency (%)*	*n*	*Reference*
Australia-Queensland	4.5	110	[75]
Australia-North Territory	0.7	132	[75]
Australia-North Queensland	0.0	54	[75]
Punjab	0.0	53	[75]
Lachish, Israel	0.0	695	[75]
Egypt XX Dynasty	1.8	379	[75]
Egypt XXI Dynasty	2.7	75	[75]
Egypt Pre-dynastic	0.0	60	[75]
Egypt Middle Kingdom	0.0	182	[75]
Egypt Late period Giza	0.0	50	[75]
Canary Islands Interior	0.0	45	[84]
Canary Islands Highlands	0.9	226	[86]
Nubia-Jebel Moya	0.0	32	[75]
Nubia-Historic	1.2	431	[87]
Nubia-Kerma	0.4	224	[87]
Ashanti	0.0	56	[75]
Khoisan	0.0	123	[88]
Ayalan, Equador	2.9	103	[75]
Botocudo, Brazil	2.5	40	[80]
Cerca Grande, Brazil	2.0	50	[80]
**Low Latitude (<30°) "wet"**			n = 30
Hawai-Oahu	0.0	1063	[75]
Hawaii-Mokapu	13.2	49	[75]
Hawaii-pooled	20.3	148	[75]
New Britain	0.0	85	[75]
New Ireland	0.0	53	[75]
Solomon Islands	0.0	50	[75]
New Caledonia	2.9	85	[75]
New Hebrides	0.0	84	[75]
Fiji	0.0	32	[75]
Society Islands	0.0	58	[75]
Lesser Sundas	0.0	45	[75]
Easter Island	8.6	64	[75]
Marquesas	2.8	51	[75]
Marquesas	18.2	36	[75]
Southern Cook Islands	3.8	52	[89]
Samoa	25	38	[89]
Island Melanesia	3.1	32	[89]
Duff Islands	6.1	59	[89]
New Guinea South Coast	3.3	95	[88]
New Guinea North/Melanesia	3.6	44	[88]
Canary Islands Galdár	64.7	34	[90]
Canary Islands Coast	40.2	97	[86]
Canary Islands fishing	8.6	105	[84]
Corondó, Brazil	0.0	32	[80]
Guaraguaçu, Brazil	13.3	30	[80]
Moro do Ouro, Brazil	18.9	37	[80]
Rio Comprido, Brazil	54.8	31	[80]
Base Aérea, Brazil	22.2	36	[80]
Tapera, Brazil	28.6	70	[80]
Cabeçuda, Brazil	43.2	74	[80]

Site	*Frequency (%)*	*n*	*Reference*
**Middle Latitude (30° - 45°) "dry"**			n = 19
Australia-Murray Valley	27.9	476	[75]
Australia-Murray Valley	21.2	99	[75]
Tasmania	4.8	62	[75]
Tasmania	9.0	67	[75]
Jomon, Japan	18.7	542	[89]
Yayoi, Japan	18.9	90	[89]
Çayönü, Turkey	17.5	97	[91]
Hopewell Mounds, IL	34.1	41	[75]
Klunk II, IL	34.0	78	[75]
Woodland, IL	2.0	50	[75]
Texas pooled	10.3	348	[92]
Pecos Pueblo, NM	2.4	500	[75]
Gran Quivira, NM	3.0	35	[75]
Grasshopper, AZ	0.0	161	[75]
Point of Pines, AZ	4.9	82	[75]
Turkey Creek, AZ	0.0	104	[75]
Pyramid Lake, NV	0.3	59	[75]
North Chile highland	0.0	549	[78]
North Chile valley	2.3	264	[78]
**Middle Latitude (30° - 45°) "wet"**			n = 15
Iron Gates pooled	29.4	126	[82]
Indian Knoll, KY	34.9	404	[76]
Isola Sacra	12.5	957	[81]
Isola Sacra	31.3	48	[93]
Velia	18.6	348	[81]
Muge pooled	19.4	72	[82]
Sado pooled	9.1	33	[82]
Vlasac Iron Gates	34.2	38	[79]
Santa Rosa Island early	8.1	62	[77]
Santa Rosa Island middle	13.9	72	[77]
Santa Rosa Island late	9.6	73	[77]
North Chile fertile coast	30.6	284	[78]
North Chile dry coast	30.8	52	[78]
Argentine wetlands	6.3	176	[94]
Körtik Tepe	48.1	81	[95]
**High Latitude (>45°) "dry"**			n = 18
North China	0.0	100	[75]
Ainu Hokkaido	1.6	128	[75]
Stuttgart-Mülhausen	5.0	60	[82]
Gurgy, France	2.1	48	[82]
Vaud-Valais	0.0	94	[82]
Southeast Scotland	0.0	50	[75]
York, UK	0.0	52	[75]
Hythe, UK	0.0	50	[75]
Salish interior	3.4	87	[75]
Inuit Yukon	2.0	50	[75]
Inuit St. Lawrence	2.0	50	[75]
Arikara/Mandan	21.1	109	[96]
Arikara-Crow Creek	2.3	613	[75]
Arikara-Mandan	8.8	34	[75]
Mandan	4.4	45	[75]

Site	*Frequency (%)*	*n*	*Reference*
Tierra-del-Fuego inland	1.9	53	[83]
Franzhausen	20.0	375	[97]
Mikulčice pooled	6.6	882	[98]
**High Latitude (>45°) “wet”**			n = 16
Hebrides	0.0	50	[75]
Shetlands	0.0	50	[75]
Iceland	0.0	82	[75]
Greenland	0.0	51	[75]
Inuit coastal	0.0	50	[75]
Pre-Aleut, AK	0.0	47	[75]
Aleut, AK	0.0	50	[75]
Salish coastal	6.5	107	[75]
Koskimo, BC	0.8	143	[75]
Site	*Frequency (%)*	*n*	*Reference*
Cowichan, BC	2.1	117	[75]
Haida, BC	0.0	36	[75]
Chinook, WA/OR	27.7	83	[75]
Tierra-del-Fuego coastal	9.1	55	[83]
Lower Volga Bronze Age	2.8	143	[99]
Na Pískách Old Slavonic	1.8	893	[100]
Vysoká zahrada Old Slavonic	1.5	129	[100]
