# Supplementary material for: External auditory exostoses among western Eurasian late Middle and Late Pleistocene humans
Source: PLoS One. 2019 Aug 14;14(8):e0220464. doi: 10.1371/journal.pone.0220464 (PMC6693685; doi:10.1371/journal.pone.0220464)
Supplement: S3 Table — (PDF) [file pone.0220464.s003.pdf]

External auditory exostoses among western Eurasian late Middle and Late Pleistocene humans

Supplementary Information

Erik Trinkaus,[1] Mathilde Samsel,[2] and Sébastien Villotte[3]

[1] Department of Anthropology, Washington University, Saint Louis MO 63130, USA. [2] UMR5199 PACEA, Université de Bordeaux, Bâtiment B8, Allée Geoffroy Saint Hilaire CS 50023, 33615 Pessac, France. [3] CNRS, UMR5199 PACEA, Bâtiment B8, Allée Geoffroy Saint Hilaire, 33615 Pessac, France

S1 Later Pleistocene and Recent Human Auditory Exostoses

Table S3. Distributions of external auditory exostosis grades among the recent human samples providing grades of severity.

	Grade 0	*Grade 1*	*Grade 2*	*Grade 3*	*n*	*Ref*
Vaud/Valais (pooled) ("dry")[1,2]	100%	--	--	--	83	[82]
Khoisan ("dry")	100%	--	--	--	123	[88]
Gurgy ("dry")[2]	97.1%	2.9%	--	--	35	[82]
Stuttgart-Mülhausen ("dry")[2]	95.6%	4.4%	--	--	45	[82]
New Guinea coast (south) ("wet")	96.7%	--	3.3%	--	92	[88]
Melanesian (north) ("wet")	95.3%	4.7%	--	--	43	[88]
Santa Rosa Island (pooled) ("wet")	89.8%	7.2%	2.4%	0.6%	166	[77]
Chile-Late Period ("wet")	78.2%	11.9%	5.9%	4.0%	101	[77]
Muge (pooled) ("wet")[2]	76.0%	22.0%	2.0%	--	50	[82]
Iron Gates (pooled) ("wet")[2]	75.2%	20.8%	4.0%	--	101	[82]
Chile-Archaic Period ("wet")	72.3%	22.3%	4.3%	1.1%	94	[78]
Isola Sacra ("wet")	68.8%	20.8%	6.3%	4.2%	48	[93]
Chile-Formative ("wet")	61.3%	21.3%	12.0%	5.3%	75	[78]
Körtik Tepe ("wet")	51.9%	34.6%	4.9%	8.6%	81	[95]
"Dry" average	98.2%	1.8%	0.0%	0.0%		
"Wet" Average	76.6%	16.6%	4.5%	2.4%		

[1] See Table S2 for an explanation and a justification of "wet'/"dry" attributions.
[2] Data from the side with the largest sample.
