# Supplementary material for: External auditory exostoses among western Eurasian late Middle and Late Pleistocene humans
Source: PLoS One. 2019 Aug 14;14(8):e0220464. doi: 10.1371/journal.pone.0220464 (PMC6693685; doi:10.1371/journal.pone.0220464)
Supplement: S1 File — (PDF) [file pone.0220464.s004.pdf]

External auditory exostoses among western Eurasian late Middle and Late Pleistocene humans

Supplementary Information

Erik Trinkaus,[1] Mathilde Samsel,[2] and Sébastien Villotte[3]

[1] Department of Anthropology, Washington University, Saint Louis MO 63130, USA. [2] UMR5199 PACEA, Université de Bordeaux, Bâtiment B8, Allée Geoffroy Saint Hilaire CS 50023, 33615 Pessac, France. [3] CNRS, UMR5199 PACEA, Bâtiment B8, Allée Geoffroy Saint Hilaire, 33615 Pessac, France

S2 Acknowledgments

V.S. Sparacello provided EAE observations from scans of the Arene Candide, San Teodoro and Villabruna auditory canals. Access to the Arene Candide scan data was provided by the Soprintendenza Archeologia, Belle Arti e Paesaggio per la città metropolitana di Genova e le province di Imperia, La Spezia e Savona; the data originally appeared in Sparacello et al. [43] and its collection was funded by Marie-Curie European Union COFUND/Durham Junior Research Fellowship (EU grant agreement 267209) and by the Wolfson Institute for Health and Wellbeing, Durham, UK. Photographs of the Spy 1 and 10 temporal bones were provided by H. Rougier, and detailed images of the Moča 1 auditory meatus were provided by A. Šefčáková. CT scans of the Oberkassel 1 and 2 crania were provided by J.J. Hublin and R. Schmitz. Curators, colleagues and institutions too numerous to acknowledge individually have provided access to original fossil specimens. To all we are grateful.

S3 Supplementary Information References

1. Rougier H. Étude Descriptive et Comparative de Biache-Saint-Vaast 1 (Biache-Saint-Vaast, Pas-de-Calais, France). Thèse de Doctorat, Université de Bordeaux 1; 2003. Available from: https://tel.archives-ouvertes.fr/tel-00475380.
2. Guipert G, Lumley MA de, Tuffreau A, Mafart B. A late Middle Pleistocene hominid: Biache-Saint-Vaast 2, north France. C R Palevol. 2011; 10:21-33. doi.10.1016/j.crpv.2010.10.006.
3. Condemi S. Les Néandertaliens de La Chaise. Paris: Comité des Travaux Historiques et Scientifiques; 2011.
4. Vlček E. Fossile Menschenfunde von Weimer-Ehringsdorf. Stuttgart: Konrad Theiss Verlag; 1993.
5. Dean D, Hublin JJ, Holloway R, Ziegler R. On the phylogenetic position of the pre-Neandertal specimen from Reilingen, Germany. J Hum Evol. 1998; 34:485-508.
6. Suzuki H, Takai F (eds). The Amud Man and his Cave Site. Tokyo: Academic Press of Japan; 1970.
7. Boule M. L'homme fossile de La Chapelle-aux-Saints. Ann Paléontol. 1911-13; 6:111-72; 7:21-56, 85-192; 8:1-70.
8. Rendu W, Beauval C, Crevecoeur I, Bayle P, Balzeau A, Bismuth T, et al. Evidence supporting an intentional Neandertal burial at La Chapelle-aux-Saints. Proc Natl Acad Sci USA. 2014; 111:81-6. doi.10.1073/pnas.1316780110.
9. Heim JL. Les hommes fossiles de La Ferrassie I: Le gisement. Les squelettes adultes (crâne et squelette du tronc). Arch Inst Paléontol Hum. 1976; 35:1-331.
10. Keith A. The Antiquity of Man. London: Williams and Norgate; 1915.

11. Sergi S. Il Cranio Neandertaliano del Monte Circeo (Circeo I). Rome: Accademia Naziolane dei Lincei; 1974.
12. Smith FH. 1976. The Neandertal remains from Krapina. A descriptive and comparative study. Dept Anthropol, Univ Tenn, Rep Invest. 15:1-359.
13. Radovčić J, Smith FH, Trinkaus E, Wolpoff MH. The Krapina Hominids: An Illustrated Catalog of the Skeletal Collection. Zagreb: Mladost Publishing House; 1988.
14. Verna C. Les Restes Humains Moustériens de la Station Amont de La Quina (Charente, France). Thèse de Doctorat, Université de Bordeaux 1; 2006.
15. Condemi S. Les Hommes Fossiles de Saccopastore et Leurs Relations Phylogénétiques. Paris: CNRS Éditions; 1992.
16. Stewart TD. The Neanderthal skeletal remains from Shanidar Cave, Iraq: A summary of findings to date. Proc Am Phil Soc. 1977; 121(2):121-65.
17. Trinkaus E. The Shanidar Neandertals. New York: Academic; 1983.
18. Fraipont J, Lohest M. La race humaine de Néanderthal ou de Canstadt en Belgique. Recherches ethnographiques sur des ossements humains, découvertes dans des dépôts quaternaires d'une grotte à Spy et détermination de leur âge géologique. Arch Biol 1887; 7: 587-757.
19. Rougier H, Crevecoeur I, Fiers E, Hauzeur A, Germonpré M, Maureille B, et al. Collections de la Grotte de Spy: (re)découvertes et inventaire anthropologique. Notae Praehistoricae 2004; 24:181-90.
20. McCown TD, Keith A. The Stone Age of Mount Carmel II: The Fossil Human Remains from the Levalloiso-Mousterian. Oxford: Clarendon Press; 1939.
21. Grün R, Stringer C. Tabun revisited: revised ESR chronology and new ESR and U-series analyses of dental material from Tabun C1. J Hum Evol 2000; 39:601–12. doi.10.1006/jhev.2000.0443.
22. Vandermeersch B. Les Hommes Fossiles de Qafzeh (Israël). Paris: CNRS; 1981.
23. Valladas H, Reyes JL, Joron JL, Valladas G, Bar-Yosef O, Vandermeersch B. Thermoluminescence dating of Mousterian "Proto-Cro-Magnon" remains from Israel and the origin of modern man. Nature 1988; 331:614-6.
24. Stringer CB, Grün R, Schwarcz HP, Goldberg P. ESR dates for the hominid burial site of Es-Skhul in Israel. Nature 1989; 338:756–8.
25. Villotte S, Samsel M, Sparacello V. The paleobiology of two adult skeletons from Baousso da Torre (Bausu da Ture) (Liguria, Italy): implications for Gravettian lifestyle. C R Palevol. 2017; 16:462-73. doi.org/10.1016/j.crpv.2016.09.004.
26. Jelínek J, Pelíšek J, Valoch K. Der fossile Mensch Brno II. Anthropos. 1959; 9:5-30.
27. Pettitt PB, Trinkaus E. Direct radiocarbon dating of the Brno 2 Gravettian human remains. Anthropol (Brno). 2000; 38:149-50.
28. Rainer F, Simonescu I. Sur le premier crâne d'homme Paléolithique trouvé en Roumanie. Anaele Academiei Romane Mem Secţ Ştiinţ. 1942; Ser III, 17:489-503.
29. Soficaru A, Petrea C, Doboş A, Trinkaus, E. The human cranium from the Peştera Cioclovina Uscată, Romania: Context, age, taphonomy, morphology and paleopathology. Curr Anthropol. 2007; 48:611-9.
30. Broca P. Sur les crânes et ossements des Eyzies. Bull Mém Soc Anthropol Paris. 1868; Série II, 3:350-92.
31. Vallois HV, Billy G. Nouvelles recherches sur les hommes fossiles de l'Abri de Cro-Magnon. L'Anthropol. 1965; 69:47-74, 249-72.
32. Henry-Gambier D. Les fossiles de Cro-Magnon (Les Eyzies-de-Tayac, Dordogne): nouvelles données sur leur position chronologique et leur attribution culturelle. Bull Mém Soc Anthropol Paris. 2002; 14:89-112.
33. Trinkaus E, Svoboda JA (eds). Early Modern Human Evolution in Central Europe: The People of Dolní Věstonice and Pavlov. New York: Oxford University Press; 2006.
34. Teschler-Nicola M (ed). Early Modern Humans at the Moravian Gate: The Mladeč Caves and their Remains. Vienna: Springer; 2006.

35. Doboş A, Soficaru A, Trinkaus E. The Prehistory and Paleontology of the Peştera Muierii, Romania. Étud Rech Archéol Univ Liège. 2010; 124:1-122.
36. Trinkaus E, Constantin S, Zilhão J (eds). Life and Death at the Peştera cu Oase. A Setting for Modern Human Emergence in Europe. New York: Oxford University Press; 2013.
37. Billy G. Étude anthropologique des restes humains de l'Abri Pataud. Bull Am Sch Prehist Res. 1975; 30:201-61.
38. Matiegka J. Homo předmostensis. Fosilní člověk z Předmostí na Moravě I. Lebky. Prague: Česká Akademie Věd a Umění; 1934.
39. Velemínská J, Brůžek J. Early Modern Humans from Předmostí near Přerov, Czech Republic. A New Reading of Old Documentation. Prague: Academia; 2008.
40. Trinkaus E, Buzhilova AP, Mednikova MB, Dobrovolskaya MV. The People of Sunghir: Burials, Bodies and Behavior in the Earlier Upper Paleolithic. New York: Oxford University Press; 2014.
41. Henry-Gambier D, Beauval C, Airvaux J, Aujoulat N, Baratin JF, Buisson-Catil J. New hominid remains associated with Gravettian parietal art (Les Garennes, Vilhonneur, France). J Hum Evol. 2007; 53:747-50. doi:10.1016/j.jhevol.2007.07.003.
42. Sparacello VS. Morphosource media M8553-10775. doi:10.17602/M2/M10775. Available from: http://www.morphosource.org/Detail/MediaDetail/Show/media_id/8553.
43. Sparacello VS, Rossi S, Pettitt P, Roberts CA, Riel-Salvatore J, Formicola V. New insights on Final Epigravettian funerary behavior at Arene Candide Cave (Western Liguria, Italy). J Anthropol Sci. 2018; 96:1-24. doi.10.4436/jass.96003.
44. Sparacello VS. Morphosource media M8347-10563. doi:10.17602/M2/M10563. Available from: http://www.morphosource.org/Detail/MediaDetail/Show/media_id/8347.
45. Sparacello VS. Morphosource media M8412-10618. doi:10.17602/M2/M10618. Available from: http://www.morphosource.org/Detail/MediaDetail/Show/media_id/8412.
46. Sparacello VS. Morphosource media M8543-10764. doi:10.17602/M2/M10764. Available from: http://www.morphosource.org/Detail/MediaDetail/Show/media_id/8543.
47. Sparacello VS. Morphosource media M8541-10762. doi:10.17602/M2/M10762. Available from: http://www.morphosource.org/Detail/MediaDetail/Show/media_id/8541.
48. Chauvière FX (ed). La grotte du Bichon: un site préhistorique des montagnes neuchâteloises: Archéologie neuchâteloise. 2008; 42.
49. Testut L. Recherches anthropologiques sur le squelette quaternaire de Chancelade. Bull Soc Anthropol Lyon. 1889; 8:131-246.
50. Barshay-Szmidt C, Costamagno S, Henry-Gambier D, Laroulandie V, Pétillon JM, Boudadi-Maligne M, et al. New extensive focused AMS 14 C dating of the Middle and Upper Magdalenian of the western Aquitaine/Pyrenean region of France (ca. 19–14 ka cal BP): Proposing a new model for its chronological phases and for the timing of occupation. Quatern Int. 2016; 414:62-91.
51. Aymard I. Étude Paléopathologique des Vestiges Humains Aziliens de l'Aven des Iboussières (Malataverne, Drôme). Diplôme d'État de Docteur en Médecine, Université de Nantes. Available at: http://www.sudoc.fr/095039856.
52. Gely B, Morand P. Les sépultures épipaléolithiques de l'aven des Iboussières à Malataverne (Drôme, France): Premiers résultats. Ardèche Archéologie 1998; 15:13-8.
53. Posth C, Renaud G, Mittnik A, Drucker DG, Rougier H, et al. Pleistocene mitochondrial genomes suggest a single major dispersal of non-Africans and a late glacial population turnover in Europe. Curr Biol. 2016; 26:827-33
54. Genet-Varcin E, Miquel M. Contribution à l'étude du squelette magdalénien de l'abri Lafaye à Bruniquel (Tarn et Garonne). L'Anthropol. 1967; 71:467-78.
55. Gambier D, Valladas H, Tisnérat-Laborde N, Arnold M, Bresson F. Datation de vestiges humains présumés du Paléolithique supérieur par la méthode du Carbone 14 en spectrométrie de masse par accélérateur. Paléo. 2000; 12:201-12.
56. Hamy ET. Description d'un squelette humain fossile de Laugerie-Basse. Bull Soc Anthropol Paris. 1874; 9:652-8.

57. Šefčáková A, Katina S, Mizera I, Halouzka R, Barta P, Thurzo M. A late Upper Palaeolithic skull from Moča (the Slovak Republic) in the context of central Europe. Acta Musei Nationalis Pragae 2011; 67B(1-2):3-24.
58. Giemsch L, Schmitz RW (eds). The Late Glacial Burial from Oberkassel Revisited. Darmstadt: Phillip von Zabern; 2016.
59. Hershkovitz I, Speirs MS, Frayer D, Nadel D, Wishbaratz S, Arensburg B. Ohalo-II H2—a 19,000-year-old skeleton from a water-logged site at the Sea of Galilee, Israel. Am J Phys Anthropol. 1995; 96:215–34.
60. Trinkaus E. The paleopathology of the Ohalo 2 Upper Paleolithic human remains: A reassessment of its appendicular robusticity, humeral asymmetry, shoulder degenerations and costal lesion. Int J Osteoarchaeol. 2018; 28(2):143-52. doi.10.1002/oa.2640.
61. Patte E. L'homme et la femme de l'Azilien de Saint-Rabier. Mém Mus Natl Hist Nat. 1968; C19:1-55.
62. Samsel M, Knüsel C, Villotte S. Réévaluation du sexe et de l'âge au décès du sujet azilien La Peyrat 5, Saint-Rabier (Dordogne, France). Bull Mém Soc Anthropol Paris. 2016; 28:213-20. doi.10.1007/s13219-016-0162-9.
63. Ferembach D. Le squelette humain Azilien de Rochereil (Dordigne). Bull Mém Soc Anthropol Paris. 1974; 2:271-91.
64. Peyrony D, Vallois HV. Le gisement et le squelette de Saint Germain-la-Rivière. Arch Inst Paléont Hum. 1972; 34:1-118.
65. D'Amore G, Di Marco S, Tartarelli G, Bigazzi R, Sineo L. Late Pleistocene human evolution in Sicily: comparative morphometric analysis of Grotta di San Teodoro craniofacial remains. J Hum Evol. 2009; 56:537-50. doi:10.1016/j.jhevol.2009.02.002.
66. Mannino M, Di Salvo R, Schimmenti V, Di Patti C, Incarbona A, Sineo L, et al. Upper Palaeolithic hunter-gatherer subsistence in Mediterranean coastal environments: an isotopic study of the diets of the earliest directly-dated humans from Sicily. J Archaeol Sci. 2011; 38:3094-100. doi:10.1016/j.jas.2011.07.009.
67. Vercellotti G, Alciati G, Richards MP, Formicola V. The Late Upper Paleolithic skeleton Villabruna 1 (Italy): A source of data on biology and behavior of a 14.000 year-old hunter. J Anthropol Sci. 2008; 86:143-63.
68. Rmoutilová R, Guyomarc'h P, Velemínský P, Šefčáková A, Samsel M, Santos F, et al. Virtual reconstruction of the Upper Palaeolithic skull from Zlatý kůň, Czech Republic: Sex assessment and morphological affinity. PLoS One. 2018; 13(8):e0201431. doi.org/10.1371/journal.pone.0201431.
69. Trinkaus E. Pathology and the posture of the La Chapelle-aux-Saints Neandertal. Am J Phys Anthropol. 1985; 67:19-41.
70. Haeusler M, Trinkaus E, Fornai C, Müller J, Bonneau N, Boeni T, et al. Morphology, pathology and the vertebral posture of the La Chapelle-aux-Saints Neandertal. Proc Natl Acad Sci USA. 2019; 116(11):4923-7. doi.org/10.1073/pnas.1820745116.
71. Trinkaus E, Villotte S. External auditory exostoses and hearing loss in the Shanidar 1 Neandertal. PLoS ONE. 2017; 12(10):e0186684. doi.10.1371/journal.pone.0186684.
72. Crubézy E, Trinkaus E. Shanidar 1: A case of hyperostotic disease (DISH) in the Middle Paleolithic. Am J Phys Anthropol. 1992; 89:411-20. doi.10.1002/ajpa.1330890402.
73. Smith FH. The Neandertal remains from Krapina. A descriptive and comparative study. Dept Anthropol, Univ Tenn, Rep Invest. 1976; 15:1-359.
74. Wolpoff MH. The Krapina dental remains. Am J Phys Anthropol. 1979; 50:67-114.
75. Kennedy GE. The relationship between auditory exostoses and cold water: a latitudinal analysis. Am J Phys Anthropol. 1986; 71(4):401-15.
76. Snow CE. Indian Knoll Skeletons of Site Oh 2, Ohio County, Kentucky. Univ Kentucky Rep Anthropol. 1948; 4(3-II):371-554.

77. Kuzminsky SC, Erlandson JM, Xifara T. External auditory exostoses and its relationship to prehistoric abalone harvesting on Santa Rosa Island, California. Intl J Osteoarchaeol. 2016; 26:1014-23. doi.10.1002/oa.2512.
78. Standen VG, Arriaza B. Santoro CM. External auditory exostosis in prehistoric Chilean populations: a test of the cold water hypothesis. Am J Phys Anthropol. 1997; 103(1):119-29.
79. Frayer DW. Auditory exostoses and evidence for fishing at Vlasac. Curr Anthropol. 1988; 29(2):346-9.
80. Okamura MMM, Boyadjian CHC, Eggers S. Auditory exostoses as an aquatic activity marker: A comparison of coastal and inland skeletal remains from tropical and subtropical regions of Brazil. Am J Phys Anthropol. 2007; 132:558-67. doi.10.1002/ajpa.20544.
81. Crowe F, Sperduti A, O'Connell TC, Craig OE, Kirsanow K, Germoni P, et al. Water-related occupations and diet in two Roman coastal communities (Italy, first to third century AD): correlation between stable carbon and nitrogen isotope values and auricular exostosis prevalence. Am J Phys Anthropol. 2010; 142(3):355-66. doi.10.1002/ajpa.21229.
82. Villotte S, Stefanović S, Knüsel CJ. External auditory exostoses and aquatic activities during the Mesolithic and the Neolithic in Europe: results from a large prehistoric sample. Anthropol (Brno). 2014; 52(1):73-89.
83. Ponce P, Ghidini G, González-José R. External auditory exostosis "at the end of the world": the southernmost evidence according to the latitudinal hypothesis. Brit Archaeol Rep. 2008; S1743: 101–7.
84. Arnay-de-la-Rosa M, González-Reimers E, Velasco-Vázquez J, Santolaria-Fernández F. Auricular exostoses among the prehistoric population of different islands of the Canary archipelago. Ann Otol Rhinol Laryngol. 2001; 110(11):1080-3. doi.org/10.1177/000348940111001117.
85. Hurst W, Bailey M, Hurst B. Prevalence of external auditory canal exostoses in Australian surfboard riders. J Laryngol Otol 2004; 118:348-31. doi.org/10.1258/002221504323086525.
86. Velasco-Vázquez J, Betancor-Rodriguez A, Arnay-de-la-Rosa M, Gonzalez-Reimers E. Auricular exostoses in the prehistoric populaion of Gran Canaria. Am J Phys Anthropol. 2000; 112:49-55.
87. Godde K. An examination of proposed causes of auditory exostoses. Int J Osteoarchaeol. 2010; 20:486-90. doi: 10.1002/oa.1058.
88. Ponzetta MT, Hauser G, Vienna A. Auditory hyperostosis and the environment: an update. Int J Anthropol. 1997; 12(2):29-42.
89. Katayama K. Auditory exostoses among ancient human populations in the Circum-Pacific area: Regional variation in the occurrence and its implications. Anthropol Sci. 1998; 106(4):285-96.
90. Dutour O, Onrubia-Pintado J. Interactions homme-environnement océanique pendant la préhistoire récente des Iles Canaries: nouvelles données paléoanthropologiques de la région de Galdar (Grande Canarie). C R Acad Sci Paris Série III, 1991; 313:125-30.
91. Özbek M. Auditory exostoses among the prepottery Neolithic inhabitants of Çayönü and Aşikl, Anatolia; its relation to aquatic activities. Int J Paleopathol. 2012; 2:181-6. doi.org/10.1016/j.ijpp.2012.10.004.
92. Goldstein MS. Skeletal pathology of early Indians in Texas. Am J Phys Anthropol. 1957; 15(3):299-311.
93. Manzi G, Sperduti A, Passarello P. Behavior-induced auditory exostoses in Imperial Roman society: Evidence from coeval urban and rural communities near Rome. Am J Phys Anthropol. 1991; 85:253-60.
94. Mazza B. Auditory exostoses in Pre-Hispanic populations of the Lower Paraná wetlands, Argentina. Int J Osteoarchaeol. 2016; 26:420-30. doi.10.1002/oa.2432.
95. Koruyucu MM, Şahin FS, Delibaş D, Erdal ÖD, Benz M, Özkaya V. Auditory exostosis: Exploring the daily life at an early sedentary population (Körtik Tepe, Turkey). Int J Osteoarchaeol. 2018; 28:615-25. doi.10.1002/oa.2674.
96. Gregg JB, McGrew RN. Hrdlička revisited (external auditory canal exostoses). Am J Phys Anthropol. 1970; 33:37-40.

97. Wiltschke-Schrotta K. Das frühbronzezeitliche Gräberfeld von Franzhausen I, Niederösterreich 3. Untersuchung des epigenetischen Merkmale. Anthropol Anz. 1992; 50(1/2):27-49.
98. Velemínský P, Dobisíková M, Stránská P, Velemínská J. Biological diversity of non-metric traits in the great Moravian population – the comparison of the Mikulčice power centre and its hinterland. In: Veleminský P, Poláček L, editors. Studien zum Burgwall von Mikulčice VIII. Brno: Archeologický Ústav Akademie Věd České Republiky; 2008. pp. 265-304.
99. Pererva EV, Djachenko AN. A stress or migration marker (a study of the auditory canal exostosis in the skeleton material of the Middle Bronze Age population from the lower Volga region burial grounds) (in Russian). Anthropology 2017; 39(4):61-78. doi.10.20874/2071-0437-2017-39-4-061-078.
100. Fojtová M. An analysis of epigenetic traits of on the skeletal remains from the Old Slavonic populations from Dolní Věstonice, Czech Republic. Anthropol (Brno). 2007; 45(1):81-90.
